# Effects of Coincubation With Crystalloids or Medications on Canine Packed Red Blood Cells: An In Vitro Evaluation

**DOI:** 10.1111/vec.70109

**Published:** 2026-05-14

**Authors:** Christopher K. Smith, Deanna M. W. Schaefer, Michael M. Fry, Lisa Neufang, Stephen A. Kania, Xiaojuan Zhu, Stephanie Kleine, Reza Seddighi

**Affiliations:** ^1^ Department of Small Animal Clinical Sciences University of Tennessee Knoxville Tennessee USA; ^2^ Department of Biomedical and Diagnostic Sciences University of Tennessee Knoxville Tennessee USA; ^3^ Office of Information Technology University of Tennessee Knoxville Tennessee USA; ^4^ Department of Large Animal Clinical Sciences University of Tennessee Knoxville Tennessee USA

**Keywords:** fentanyl, propofol, saline, transfusion, vasopressor

## Abstract

**Objective:**

To investigate the effects of coincubation of various solutions and medications with canine packed red blood cells (pRBCs).

**Design:**

Prospective in vitro study.

**Experimental Units:**

Ten commercially prepared bags of canine pRBCs and fresh frozen plasma (FFP).

**Interventions:**

pRBCs were coincubated with normal saline solution (NSS), 5% dextrose (D5W), Normosol‐R, fentanyl, propofol, norepinephrine, dopamine, or same‐donor FFP, as a control. Two pRBC storage times (<7 and 28–35 days) and two incubation times (3 and 30 min) were used.

**Measurements and Main Results:**

CBC, light microscopy, methemoglobin assay, and osmotic fragility (OF) testing were performed to assess for hemolysis (decreased RBC count, decreased cellular hemoglobin), RBC swelling (increased mean cell volume [MCV], increased OF), and oxidant injury (increased methemoglobin, Heinz body formation). Results were analyzed by mixed‐effect ANOVA to detect significant (*p* < 0.05) differences; results were also compared with the coefficient of variation of each test method to detect differences exceeding analytical imprecision. All results were compared with coincubation with same‐donor FFP under the same conditions (storage and incubation times). NSS caused significant increases in MCV under all conditions. For >28‐day‐old blood, MCV was significantly increased with dextrose, norepinephrine, dopamine, and fentanyl. OF was mildly but significantly increased with SAL, norepinephrine, dopamine, and fentanyl. Coincubation with propofol resulted in many changes consistent with lipid emulsion artifact, impeding the detection of true additive effects.

**Conclusions:**

Under the most ideal conditions tested (short coincubation of <7‐day‐old pRBCs), most additives had minimal deleterious effects on RBCs. However, NSS caused mild RBC swelling under all conditions. Mild RBC swelling and increased OF were also observed with most additives in older blood. The propofol lipid emulsion causes analytical interference that confounded the interpretation of results. Further investigation into the safety and efficacy of coadministration of RBCs with other products, including NSS, is indicated.

**Abbreviations**
CV: coefficient of variation
D5W: dextrose 5% in water
FFP: fresh frozen plasma
HGB: hemoglobin
MCV: mean cell volume
MPC: mean platelet component
NSS: normal (0.9%) saline solution
OF: osmotic fragility
PLT: platelet concentration
pRBC: packed red blood cell

## 1 Introduction

In the United States, approximately 16 million blood components are transfused to people each year [1]. National data on the number of veterinary transfusions have not been published, but one veterinary teaching hospital reports around 600 transfusions per year [2]. The goal of blood product administration is to improve a patient's clinical condition by supplying components in which they are deficient. For example, packed red blood cells (pRBCs) are commonly administered in anemic patients to improve oxygen‐carrying capacity [3]. With recent recommendations for earlier use of blood products in various resuscitation models, veterinary blood product transfusions are likely to increase [4, 5].

Blood product administration practices should employ evidence‐based methods that maximize efficacy and minimize patient morbidity. The American Association of Blood Banks currently recommends infusion of human blood products only through a dedicated IV catheter or a dedicated lumen of a multi‐lumen catheter with concurrent administration of no other fluids or medications other than normal saline solution (NSS) [6]. This practice stems from concerns that concurrently administered solutions or medications will deleteriously affect the transfused blood products, reducing their efficacy or exacerbating patient morbidity. However, requiring a dedicated catheter for blood product transfusions, particularly in critically ill patients, may increase the burden on both medical professionals and patients. Patient factors, including small size, trauma to sites intended for catheter placement, obesity, and edema, can all hinder IV accessibility and ultimately delay treatment, which may increase morbidity and mortality [7]. Furthermore, although the American Heart Association recommends central venous catheters for ideal resuscitation [8], the use of these lines is associated with adverse events, with the development complications reported in approximately 15% of patients [9]. Thus, the recommendation and practice of providing a dedicated catheter for transfusion of blood products warrant evidence‐based justification. A recent in vitro investigation using human blood indicated that pRBCs incubated with isotonic crystalloids or catecholamines did not deleteriously alter them [10]. The study authors questioned the practice of a dedicated catheter for transfusions and advocated for reducing additional IV catheterization. Currently, there are no similar published studies using canine RBCs.

A recent survey of veterinary referral and teaching hospitals showed approximately 75% of hospitals use a dedicated IV catheter for blood product administration, with the remaining 25% only allowing concurrent administration of isotonic crystalloids [11]. Various sources in the veterinary literature (e.g., textbooks [12, 13], review articles [14], and manuals [15]) support the practice of using a dedicated catheter for administration of blood products, without strong evidence based on controlled study. Although blood product administration guidelines have been established in human medicine [6], no such clear guidelines exist in the veterinary literature. Therefore, current veterinary practice is largely based on extrapolation from human guidelines, opinion, or conjecture [16].

The main aim of the current study was to evaluate how canine pRBCs are affected when coincubated with various additives, including common peri‐anesthetic medications or crystalloid solutions. Secondary aims of the study were to evaluate these effects with different ages of pRBCs and coincubation times. The null hypothesis was that, under the same storage and incubation time conditions, there would be no differences in test results between any additive and a same‐donor fresh frozen plasma (FFP) negative control.

## 2 Materials and Methods

All work, aside from osmolality measurement (see below), was performed at the University of Tennessee College of Veterinary Medicine.

### 2.1 Blood Products and Experimental Design

In vitro experiments were performed using 10 bags of same‐donor canine pRBCs and FFP purchased from a blood bank^1^, and both products were stored in citrate–phosphate–dextrose–adenine solution. After receipt of pRBCs and FFP, the bags were stored until use in a 4°C refrigerator and a −20°C freezer, respectively. Treatments consisted of incubating pRBCs with different additives (drugs or crystalloid solutions, or autologous FFP as a negative control) under four conditions (two pRBC storage times and two incubation times). Two ages of blood, <7 days (younger) and 28–35 days (older) from the collection date, were evaluated to assess the effect of age of blood on results. Each pair was used for both 3‐ and 30‐min incubation conditions within a 24‐h period.

### 2.2 Additives

Specific additives used, drug concentrations, and volume of crystalloid or drug added to pRBCs are shown in Table 1. These additives were chosen because they are in common use clinically and would be desirable for coadministration with blood products if it could be done safely. Drug amounts reflect clinical dosages.

**TABLE 1:** Additive concentrations and volumes combined with pRBCs.

Additive	Volume added to 1 mL pRBCs
Normosol‐R^9^	1 mL
D5W^10^	1 mL
NSS^11^	1 mL
FFP^1^	1 mL
Propofol^12^ (10 mg/mL)	360 µL
Fentanyl^13^ (50 µg/mL)	20 µL
Dopamine^14^ (6 mg/mL)	10 µL
Norepinephrine^15^ (1 mg/mL)	6 µL

Abbreviations: D5W, dextrose 5% in water; FFP, fresh frozen plasma; NSS, normal (0.9%) saline solution; pRBCs, packed red blood cells.

### 2.3 Dosages and Coincubation Times

Two coincubation times, 3 and 30 min, were used. Three minutes approximates the time the two solutions would reside together in a jugular catheter and IV tubing (with a 5‐mL capacity) at the clinically based rates of administration. Thirty minutes significantly exceeds (10×) any amount of time the two infusions would reside together in an actual clinical scenario and was chosen as an extreme to see whether changes were detectable. A body weight of 20 kg was chosen as a mid‐range canine weight to calculate fluid and medication doses as well as rates of administration. Common peri‐anesthetic rates of administration were chosen for crystalloid administrations (5 mL/kg/h) and pRBC transfusion (10 mL/kg of pRBCs administered over 2 h). On the day of the experiment, pRBCs and the additives were pipetted into an additive‐free polypropylene vial^2^ in ratios based on the previous clinical example (Table 1). Once the vial contained both additives and pRBCs, it was allowed to mix gently on a laboratory rocker for the allotted incubation time.

### 2.4 Sample Analysis

Once the given incubation time had elapsed, the sample was analyzed using several established methods. CBCs were performed on a hematology analyzer^3^ equipped with veterinary software, using the canine setting. Methemoglobin percentage was measured on a blood gas analyzer^4^, in which each sample was first diluted with autologous FFP to create a total volume of 2 mL in order to decrease the viscosity of the sample and avoid clogging the instrument.

Blood smears were prepared two ways: after incubation of the sample with new methylene blue using routine methodology [17] to highlight any Heinz bodies present, and conventionally, using an automated stainer and conventional Romanowsky‐type stain^5^. Microscopic evaluation of these smears was performed by board‐certified veterinary clinical pathologists (D.M.W.S., M.M.F.). Osmotic fragility (OF) was assessed using an established spectrophotometric^6^ method [18]. Table 2 summarizes the evidence that was used to support deleterious effects on pRBCs and platelets after coincubation.

**TABLE 2:** Analytical results (compared with autologous FFP‐treated controls) considered as evidence of deleterious effects on RBCs or platelets.

Deleterious effect	Supportive evidence
Hemolysis	Decreased RBC count Decreased cellular HGB
RBC swelling	Increased MCV
Increased RBC osmotic fragility	Increased saline concentration at 50% lysis
Oxidant injury to RBCs	Heinz bodies identified on a blood smear stained with new methylene blue Increased methemoglobin
Increased platelet activation or platelet swelling	Decreased PLT Increased MPV Decreased MPC

Abbreviations: HGB, hemoglobin; MCV, mean cell volume; MPC, mean platelet component; MPV, mean platelet volume; PLT, platelet concentration.

### 2.5 Ancillary Experiments

Using an additional bag of FFP and other materials (all obtained from the same providers as for the initial experiments), several additional experiments were performed to address questions arising from the results of the initial experiments. Platelet concentration (PLT) in the initial experiments was often lower than the control after treatment with all additives, except propofol, leading to a hypothesis that FFP itself (the negative control) contains platelets. Additionally, the initial experiments with propofol produced cell counts that were often higher, in addition to a concentration of hemoglobin (HGB) that was consistently higher, leading to a hypothesis that propofol contains a positive interferent affecting those measurements. Lastly, mean cell volume (MCV) was consistently higher after treatment with NSS, leading to a hypothesis that NSS is hypo‐osmolar compared with canine FFP.

Additional experiments were performed to test the three new hypotheses. PLT was measured on FFP (<7 and >28 days) samples without added pRBCs, and smears of FFP were microscopically evaluated for the presence of platelets. CBCs were run on samples of propofol mixed only in FFP (<7 and >28 days) in the same dilution as the original experiments (360 µL of propofol in 1000 µL of FFP) to measure RBCs, platelets, WBC, and HGB. NSS, FFP, dextrose 5% in water (D5W), and Normosol samples were sent for osmolality testing by freezing point depression to the Cornell University Animal Health Diagnostic Laboratory.

### 2.6 Statistical Methods

The number of pRBC bags (*n* = 5) included in the initial experiments for each condition was based on a power analysis specifying 80% power to detect a 10% difference in hemolysis, an SD of 5%, a correlation of 0.30, and a significance level of 0.05, using a single‐factor repeated‐measures design [19]. The experimental design randomized the order of both the blood samples and solutions at different pRBC ages and coincubation times using complete block design with split–split plot. The design was generated using statistical software^7^.

Unless otherwise stated, all results were compared with FFP under the same condition (storage and incubation time). The four conditions were as follows: <7‐day‐old blood and 3‐min incubation; <7‐day‐old blood and 30‐min incubation; 28‐ to 35‐day‐old blood and 3‐min incubation; and 28‐ to 35‐day‐old blood and 30‐min incubation.

All continuous measurements were tested for normality using the Shapiro–Wilk test and *Q*–*Q* normality plots. Because nine variables (of 15) violated the normal distribution, the mixed‐effect ANOVA on ranks was used to determine the effects of interventions, incubation times, storage times, and their interaction by controlling for the random effect of blood bag × storage time and blood bag × storage time × incubation time. The Tukey–Kramer test was used for post hoc multiple comparison between the interventions, incubation times, and storage times, and their interaction effects. A *p*‐value of <0.05 was considered statistically significant. Commercial statistical software^8^ was used for all analyses.

The study also evaluated whether differences in results could have been attributable solely to analytical imprecision. All analytical methods have inherent imprecision (analytical imprecision), which can be estimated by determining the coefficient of variation (CV; calculated as the SD divided by the mean) of results obtained after repeated measurements of the same sample. Using CV values developed by the clinical pathology laboratory where experiments were performed as a basis for comparison, any differences that were statistically significant and also exceeded the CV (reported as analytical imprecision [%]) were considered to have been due at least in part to treatment effects.

## 3 Results

Tables 3 and 4 summarize relevant CBC and OF testing results. In addition to the continuous numeric CBC data, the hematology analyzer generated binary data (present or absent) for RBC ghosts (lysed erythrocytes), RBC fragments, and platelet clumps. Thirteen of 20 of the samples incubated with propofol were the only ones in which RBC ghosts or fragments were reported by the analyzer. To support the automated findings, Wright‐stained blood smears of 14 samples incubated with propofol were microscopically evaluated by one clinical pathologist (D.M.W.S.), including samples in which the analyzer reported neither ghost cells nor fragments (*n* = 1), reported only fragments (*n* = 5) or only ghost cells (*n* = 1), or reported both (*n* = 7). Representative photomicrographs of these samples were evaluated by a second clinical pathologist (M.M.F.), and both clinical pathologists concluded that there were no RBC fragments in any of the samples but, rather, the RBCs were commonly abnormally shaped (poikilocytosis).

**TABLE 3:** Results of CBC and OF testing on canine pRBCs coincubated with various medications for 3 min, shown as percentage difference from control treatment with autologous FFP.

Analyte									
Additive	Blood age (days)	RBC	HGB	MCV	cHGB	PLT	MPV	MPC	OF
NSS	<7			3.1%		−40.5%		23.8%	
Normosol‐R	<7					−52.0%		20.5%	
D5W	<7	−2.4%			−2.1%	−73.8%	15.6%	17.3%	
Norepinephrine	<7			2.3%		−65.7%	8.0%	20.5%	
Dopamine	<7			2.4%		−58.7%		25.9%	
Fentanyl	<7			2.3%	−1.7%	−54.9%	19.2%	24.9%	
Propofol	<7	4.1%	25.2%	21.9%	−4.4%	126.7%	−8.3%	−20.0%	14.2%
NSS	>28	4.5%	2.3%	6.3%				3.3%	4.6%
Normosol‐R	>28	3.3%	2.6%	0.9%					
D5W	>28	28.0%	2.6%		5.5%		−35.9%	21.9%	−7.6%
Norepinephrine	>28	1.5%	2.9%	4.5%					5.5%
Dopamine	>28		3.9%	4.4%				5.6%	4.7%
Fentanyl	>28		2.0%	4.7%				4.5%	4.7%
Propofol	>28	21.0%	42.8%	31.7%	8.7%	722.1%	−11.5%	−27.8%	15.3%
Analytical imprecision (%)		1.3%	1.6%	0.6%	1.5%	26.9%	4.8%	2.6%	—

*Note*: For CBC data, only those results with absolute % values exceeding the analytical imprecision for the analyte in question are shown, and results statistically significantly different from the control treatment are shaded.

Abbreviations: cHGB, cellular hemoglobin; D5W, dextrose 5% in water; FFP, fresh frozen plasma; HGB, hemoglobin; MCV, mean cell volume; MPC, mean platelet component; MPV, mean platelet volume; NSS, normal (0.9%) saline solution; OF, osmotic fragility; PLT, platelet concentration.

**TABLE 4:** Results of CBC and OF testing on canine pRBCs coincubated with various medications for 30 min, shown as percentage difference from control treatment with autologous FFP.

Analyte									
Additive	Blood age (days)	RBC	HGB	MCV	cHGB	PLT	MPV	MPC	OF
NSS	<7	−1.4%		5.0%	−4.5%	−45.0%		27.7%	
Normosol‐R	<7			1.5%	−2.1%	−79.7%	39.1%	14.7%	
D5W	<7	−2.3%		−2.4%		−91.4%	35.0%	−24.3%	
Norepinephrine	<7			3.2%		−55.5%	8.0%	24.5%	
Dopamine	<7			3.0%		−53.4%		25.0%	
Fentanyl	<7	1.6%		3.0%		−51.7%	9.5%	28.8%	
Propofol	<7		12.8%	25.5%	−8.5%		5.1%	−23.7%	14.8%
NSS	>28		1.7%	6.7%	−2.6%	1.5%			3.1%
Normosol‐R	>28			1.5%	−1.6%	−46.0%	22.6%	−6.1%	
D5W	>28			2.2%	−1.7%	−37.8%	21.9%	−4.8%	
Norepinephrine	>28			5.5%	−2.8%				3.9%
Dopamine	>28			5.6%	−2.0%				
Fentanyl	>28	−2.9%		5.5%	−5.3%		6.6%		
Propofol	>28	−2.5%	18.2%	31.2%	−12.8%		−5.9%	−29.3%	22.8%
Analytical imprecision (%)		1.3%	1.6%	0.6%	1.5%	26.9%	4.8%	2.6%	—

*Note*: For CBC data, only those results with absolute % values exceeding the analytical imprecision for the analyte in question are shown, and results significantly different from the control treatment are shaded. For OF data, only results significantly different from the control treatment are shown.

Abbreviations: cHGB, cellular hemoglobin; D5W, dextrose 5% in water; FFP, fresh frozen plasma; HGB, hemoglobin; MCV, mean cell volume; MPC, mean platelet component; MPV, mean platelet volume; NSS, normal (0.9%) saline solution; OF, osmotic fragility; PLT, platelet concentration; pRBCs, packed red blood cells.

The analyzer reported platelet clumps in fewer of the <7‐day‐old samples compared with >28‐day‐old samples (*p* = 0.00001). There were no differences based on additive or incubation time. No differences in the proportion of methemoglobin were detected, compared with control treatment with autologous FFP (data not shown). Last, no Heinz bodies were observed on any of the new methylene blue‐stained slides, which were independently reviewed by two clinical pathologists blinded to experimental conditions.

### 3.1 Ancillary Experiment Results

**FIGURE 1:**
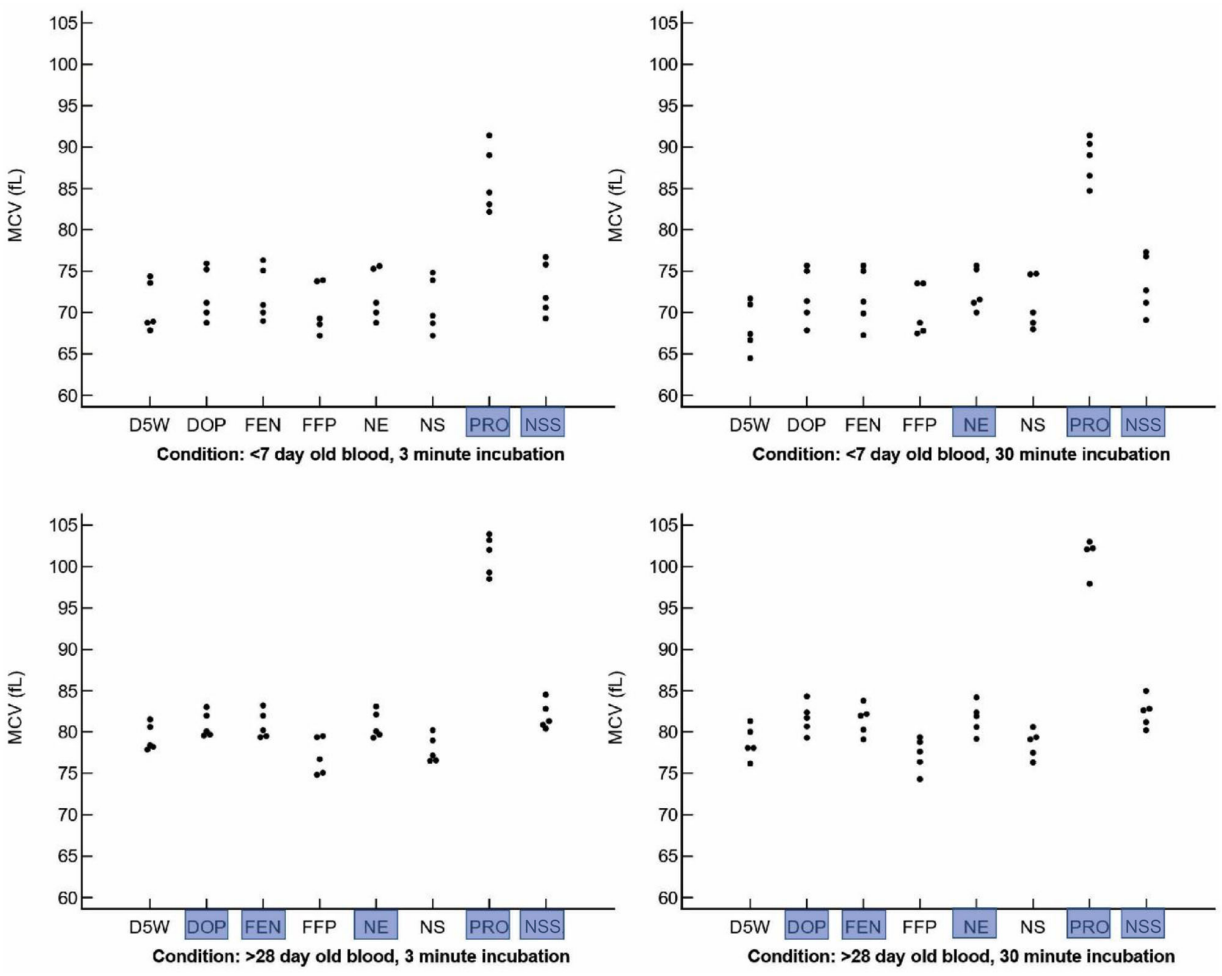
Mean cell volume (MCV) of canine red blood cells incubated with various crystalloids or medications including 5% dextrose (D5W), dopamine (DOP), fentanyl (FEN), fresh frozen plasma (FFP), norepinephrine (NE), Normosol‐R (NS), propofol (PRO) and 0.9% normal saline solution (NSS). Additives shaded are significantly different from the FFP control.

Platelet counts measured on an FFP sample <7 days old and again when it was >28 days old were 32 × 10^9^/L and 54 × 10^9^/L, respectively. Low numbers of possible platelets were microscopically identified on a smear of the sample (Figure 1). Results of experiments comparing pRBC‐free samples of FFP with those of propofol diluted in FFP are provided in the Supporting Information Table. The measured osmolality of canine FFP was higher (331 mOsm/kg H_2_O) than that of NSS, D5W, or Normosol (289, 267, and 278 mOsm/kg H_2_O, respectively).

## 4 Discussion

The current study investigated the in vitro effects of coincubating pRBCs with various additives. Overall, the null hypothesis was partially supported. Under the same storage and incubation time conditions, there were no significant differences in test results between FFP and other additives for most outcome measures. However, some significant differences were detected. The most striking findings were that NSS consistently caused RBC swelling, evidenced by an increase in MCV, and platelet counts were often higher in negative control FFP samples.

Of the four conditions tested, the short (3‐min) incubation time and the younger (<7‐day‐old) blood represent a typical amount of time that two solutions would exist coinfused in the same catheter and a model bag of blood, based on previous reports that longer storage times may lead to adverse patient outcomes [20, 21]. In this short incubation and young blood condition, and excluding propofol, only NSS caused deleterious effects on pRBCs in the form of cellular swelling (increased MCV), and this NSS effect on MCV also persisted in the other three conditions of coincubation time and storage time. Neither the underlying mechanism nor the clinical implication of this finding is clear. Mechanistically, it might involve fluid shifts in response to NSS being of relatively low osmolality compared with pRBCs, promoting water to move from the extracellular to the intraerythrocytic compartment. NSS has been shown to alter the shape of human RBCs from the normal biconcave morphology to spherical when not coincubated with an osmotic pressure–maintaining substance (e.g., glucose) or mannitol [22]. This NSS‐induced cell swelling is in contrast to what is typically observed in canine pRBCs subjected to storage in CPDA‐1 within 1 week, where there is typically a reduction in MCV, likely due to hyperosmolar conditions of CPDA‐1, followed by a gradual return in volume [23, 24]. Although MCV increased with all additives when the blood aged >28 days, NSS caused the greatest increase compared with other additives and was significantly different than FFP, suggesting the increased MCV was not solely the result of aging blood in these conditions. Differences in pH of pRBCs coincubated with NSS compared with FFP may have also played a role in the observed increases in MCV, particularly as the cells aged [25].

NSS has been shown to cause greater hemolysis during washing of human RBCs compared with plasmalyte A [26] and compared with not washing at all during neonatal extramembrane corporeal membrane oxygenation [27]. However, in the current study, although increases in OF were seen, significant changes in indicators of hemolysis (decrease in cellular HGB and RBCs) were not observed. The increase in MCV and OF caused by NSS may be due to osmolality differences between plasma and NSS. It has been suggested that IV fluids chosen for administration have the same osmolality, not osmolarity, as plasma and other extracellular fluids [28]. NSS has a theoretical osmolarity of 308 mOsm/L but an actual osmolality of 286 mOsm/L, and this disparity is due to some electrolytes being osmotically inactive [28]. The osmolality of NSS used in this study, measured by freezing point depression, was 289 mOsm/kg H_2_O.

Although pRBCs are subject to the hyperosmolar (470 mOsm/L) conditions of citrate–phosphate–dextrose–adenine solution [23], and the FFP used in the current study had a measured osmolality of 331 mOsm/kg H_2_O, the addition of a hypo‐osmolar solution such as NSS would theoretically increase cell swelling. However, this does not account for the fact that NSS, when compared with FFP, caused significant increases in MCV, while the other isotonic (Normosol, measured osmolality of 278 mOsm/kg H_2_O) or hypotonic (D5W, measured osmolality of 267 mOsm/kg H_2_O) fluids did not. Our findings add to a growing body of literature stating that NSS may not constitute a physiologic solution [29] and challenges [30] the long‐standing assumptions of NSS being compatible with blood components and the solution of choice for washing/salvaging RBCs. Because the osmolality of stored blood products is likely to be hyperosmolar to that of a patient and to NSS, it is possible that NSS is not the ideal solution to coinfuse with blood products or perform RBC washing before transfusion [26].

No significant changes to RBCs were observed when coincubated with D5W. Other studies have reported mixed effects of D5W on RBCs, with some showing an increase in hemolysis [10] while others report minimal deleterious effects [31].

Increased pRBC storage time was associated with an accentuated increase in MCV with most additives, especially NSS. This finding is consistent with other literature suggesting MCV increases with storage time [32, 33]. In dogs, this is thought to be due to equilibration to higher osmolarity or perhaps to alteration in cation transporter function due to cold storage conditions and acidification [23]. OF was increased in >28‐day‐old blood coincubated for 3 min with NSS, norepinephrine, dopamine, and fentanyl, and for 30 min with NSS and norepinephrine, suggesting that these additives may increase the likelihood of cell lysis after longer storage times. It is unclear why 30‐min coincubation with dopamine and fentanyl no longer resulted in increased OF, but because 3‐min coincubation mimics the clinical scenario of transfusion better than 30‐min incubation, the results for the shorter incubation time are more likely to be clinically relevant.

In addition to effects on RBCs, there were effects involving platelets detected in the short incubation and young blood coincubation condition. Every additive, except propofol, had lower PLT relative to the FFP‐treated negative control, five of six of which were significant: Normosol, D5W, norepinephrine, dopamine, and fentanyl. Additionally, all additives caused an increase in mean platelet component (MPC), three of six of which were significant: NSS, dopamine, and fentanyl. The explanation for the lower PLT in samples treated with these additives is not likely due to dilution, because the volume of some additives, particularly norepinephrine, was quite small. Rather, the lower PLT associated with those additives relative to FFP was likely because the FFP contained platelets, a phenomenon that has been previously documented [34] and was borne out by our ancillary experiment supporting the presence of platelets in the FFP used for the current study.

The difference in PLT between the <7‐ and >28‐day‐old FFP samples more likely reflects analytical imprecision than an actual increase in platelet numbers during the time interval between assays. PLT measurement in general is relatively imprecise. Moreover, because CV represents SD expressed as a percentage of the mean, CV may be higher in samples with lower measured values. This is relevant in this case because the laboratory CV values for PLT shown in Tables 3 and 4 were based on 20 replicate measurements of samples with a mean value of 131 × 10^9^/L (individual data not shown), but the mean PLT of the FFP sample used for the ancillary experiment was roughly one third of that. Therefore, the PLT imprecision for the FFP samples would likely have been higher.

Another factor possibly contributing to significantly lower PLT could be that some additives altered the size, distribution, or activation of platelets enough that the analyzer no longer recognized them as platelets. However, no significant effects on mean platelet volume (MPV) were observed in the short incubation, young blood condition, and the MPC pattern was the opposite of what is expected as a result of platelet activation, where MPC is based on platelet refractive index, which tends to decrease when platelets activate and release their granule contents [35]. The reason for the significant increase in MPC in samples coincubated with NSS, dopamine, and fentanyl is not clear. Last, regarding platelets, the observed effect of a lower PLT in the <7‐day‐old blood was mostly absent in the older blood, likely due to a loss of PLT in FFP over time, which would be similar to the expected decreases in PLT found in stored whole blood [36]. Evaluating platelet clumping was not part of our study design, but the analyzer generated platelet flagging data, and we evaluated a subset of Wright‐stained blood smears for platelet clumping, results that are included as supplemental data. A more complete consideration of the effects of additives on platelet clumping in pRBCs should be a component of future investigation.

Propofol caused a myriad of observed effects, such as increases in HGB, MCV, and PLT. Interpretation of these observations is problematic because distinguishing between true additive effect and analytical error is difficult. Because propofol contains a lipid vehicle, it is likely the analyzer interpreted lipid micelles to be any of the three cell lines. The analyzer used has been shown to report a falsely increased WBC count in the presence of lipemia [37]. Additionally, while RBC fragments were frequently reported in samples incubated with propofol, microscopic evaluation supported that the RBCs were abnormally shaped but not fragmented. This finding of poikilocytosis is nonspecific and can be seen in a variety of disorders. The hematology analyzer also reported lysed erythrocytes, or RBC ghosts, in some propofol‐incubated samples. The presence of lysed erythrocytes was confirmed by microscopic review in samples with and without automated detection of RBC ghosts. Lysed erythrocytes can be identified commonly in lipemic blood samples; in this case, they are attributed to the lipid vehicle of propofol [17]. Based on the presence of lysed erythrocytes, it is possible that propofol may have a detrimental effect on the RBCs, in addition to the analytical artifacts.

Propofol also increased OF in all four conditions, but again, this may have been erroneous due to the lipid interference with the spectrophotometric evaluation of the dilutions performed for OF testing, because lipids may absorb light in a range of wavelengths (300–700 nm), including the 540‐nm wavelength used for OF testing [38]. Although results are not conclusive, they support that some differences noted in coincubated propofol samples may be artifacts of the drug vehicle; however, true deleterious effects of propofol on coadministered RBCs cannot be ruled out.

This study has several limitations. One is its purely in vitro approach, which has unclear clinical relevance. A second limitation is that this study incorporated only canine pRBCs, so the applicability to pRBCs of other species is unknown. This limitation is particularly relevant given the marked differences in normal erythrocyte biochemistry among species, including major differences in intraerythrocytic electrolyte concentrations [39]. Another limitation is methodologic: it is possible that other laboratory analyses would have revealed other changes (e.g., rheologic analysis by ektacytometry [10] or microfluidics [40]). Yet another limitation is that the current study did not seek to evaluate whether coincubation of pRBCs with different drugs affected their concentrations, which might alter pharmacokinetics [10].

In conclusion, the current study found that coincubation of canine pRBCs with NSS consistently caused RBC swelling, which could be due at least in part to the relative hypo‐osmolality of NSS. No evidence was found that norepinephrine, fentanyl, dopamine, or Normosol‐R caused cellular damage when <7‐day‐old pRBCs were coincubated for 3 min, but some of these additives were associated with RBC swelling and increased OF when coincubated with >28‐day‐old pRBCs. The presence of platelets in FFP caused differences in PLT between pRBC samples treated with FFP and those treated with other additives. Propofol caused many changes that were attributable to analytical interference from the drug's lipid matrix, impeding the detection of true additive effects.

Overall, the findings of the current study suggest that it might be possible to safely coadminister some crystalloid solutions other than NSS, and some drugs, with pRBCs in dogs; that the freshest possible blood products should be used; and that the impact of coadministration of propofol with pRBCs remains unknown. Future work in this area should evaluate if coincubation of RBCs with other substances causes significant rheologic detriment, and eventually, labeled RBCs could be coinfused into live subjects with some of the substances evaluated in this work to evaluate their survivability. In addition, our results support that the practice of washing or coinfusing blood products with NSS should continue to be scrutinized, based on a growing body of evidence suggesting the deleterious effects it may have on RBCs.

## Funding

Funding was provided by the Companion Animal Fund and the Faculty Education Advancement Research Fund at the University of Tennessee College of Veterinary Medicine.

## Conflicts of Interest

The authors declare no conflicts of interest.

## Supporting information




**Supporting File 1**: vec70109‐sup‐0001‐SuppMat.pdf.

**Supporting File 2**: vec70109‐sup‐0002‐Table.docx.

## Endnotes

1 North American Veterinary Blood Bank, Manassas, VA.

2 Micro centrifuge tubes, JetBiofil Europe, Alicante, Spain.

3 Advia 2120i hematology system, Siemens Medical Solutions, Malvern, PA.

4 Advia 2120i hematology system, Siemens Medical Solutions, Malvern, PA.

5 Romanowsky stain, Wescor Aerospray, Logan UT.

6 BioSpec‐nano spectrophotometer, Shimadzu Scientific Instruments, Carlsbad, CA.

7 JMP, version Pro 15.1.0, JMP Statistical Discovery LLC, Cary, NC.

8 SAS Institute Inc., Cary, NC.

9 Normosol‐R, ICU medical, Inc., Lake Forest, IL.

10 D5W, ICU medical, Inc., Lake Forest, IL.

11 Normal Saline Solution, ICU medical, Inc., Lake Forest, IL.

12 Propofol, Zoetis Inc., Kalamazoo, MI.

13 Fentanyl, Hikma, Berkeley Heights, NJ.

14 Dopamine, Hospira Inc., Lake Forest, IL.

15 Norepinephrine, Mylan Institutional LLC, Morgantown, WV.
