# Supplementary material for: Effects of Coincubation With Crystalloids or Medications on Canine Packed Red Blood Cells: An In Vitro Evaluation
Source: J Vet Emerg Crit Care (San Antonio). 2026 May 14;36(3):308–16. doi: 10.1111/vec.70109 (PMC13350283; doi:10.1111/vec.70109)
Supplement: Supplementary file 1 — Supporting File 1: vec70109‐sup‐0001‐SuppMat.pdf. [file VEC-36-308-s002.pdf]

RBC

D5W = 5% dextrose
DOP = Dopamine
FEN = Fentanyl
FFP = Fresh frozen plasma
NE = Norepinephrine
NS = Normosol-R
PRO = Propofol
SAL = normal saline solution

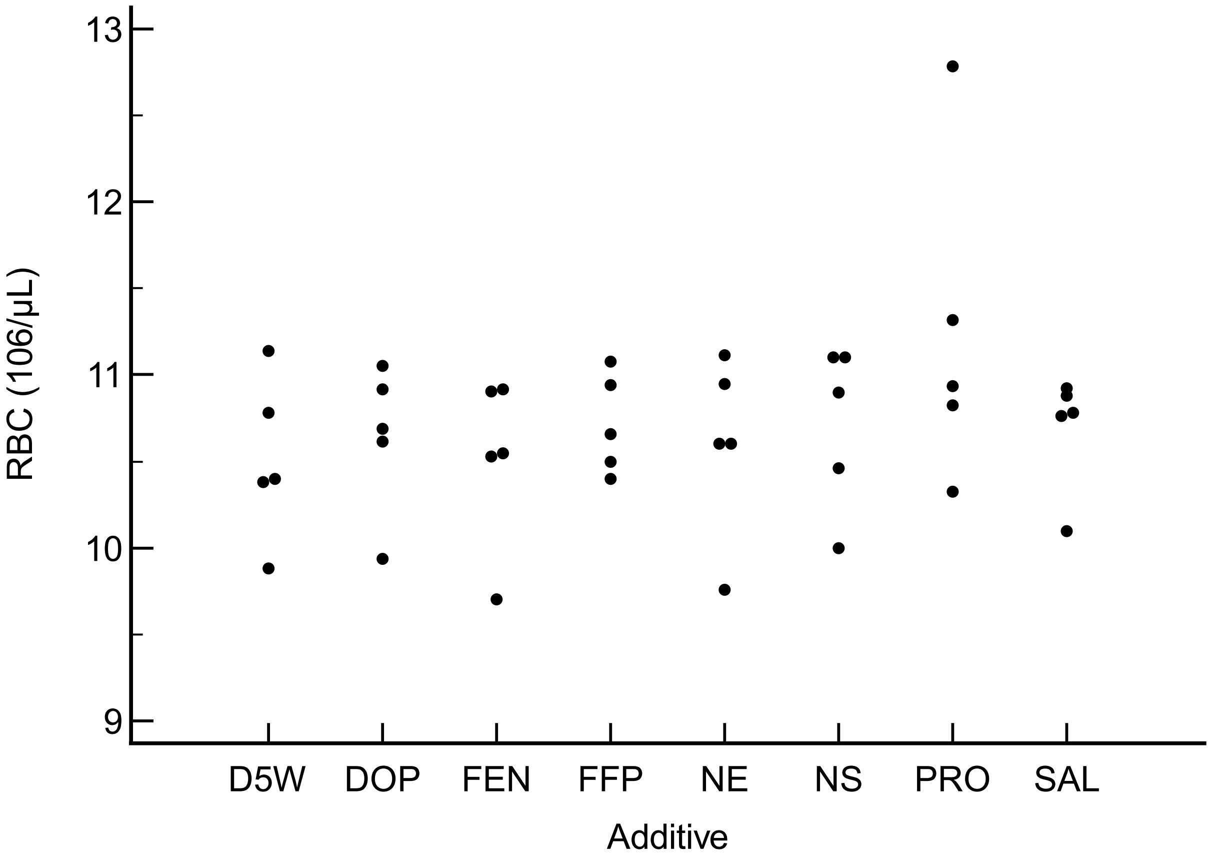

Condition: <7 day old blood, 3 min incubation

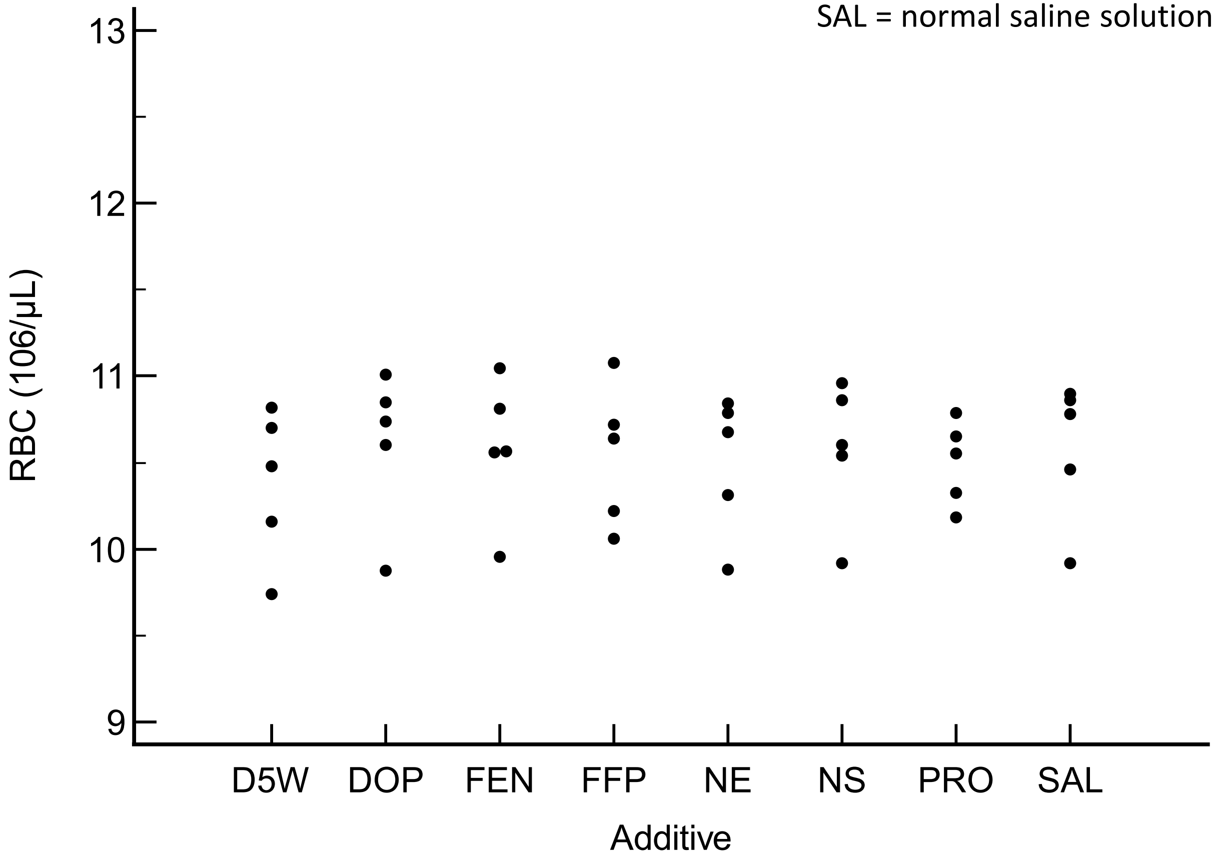

Condition: <7 day old blood, 30 min incubation

RBC

D5W = 5% dextrose
DOP = Dopamine
FEN = Fentanyl
FFP = Fresh frozen plasma
NE = Norepinephrine
NS = Normosol-R
PRO = Propofol
SAL = normal saline solution

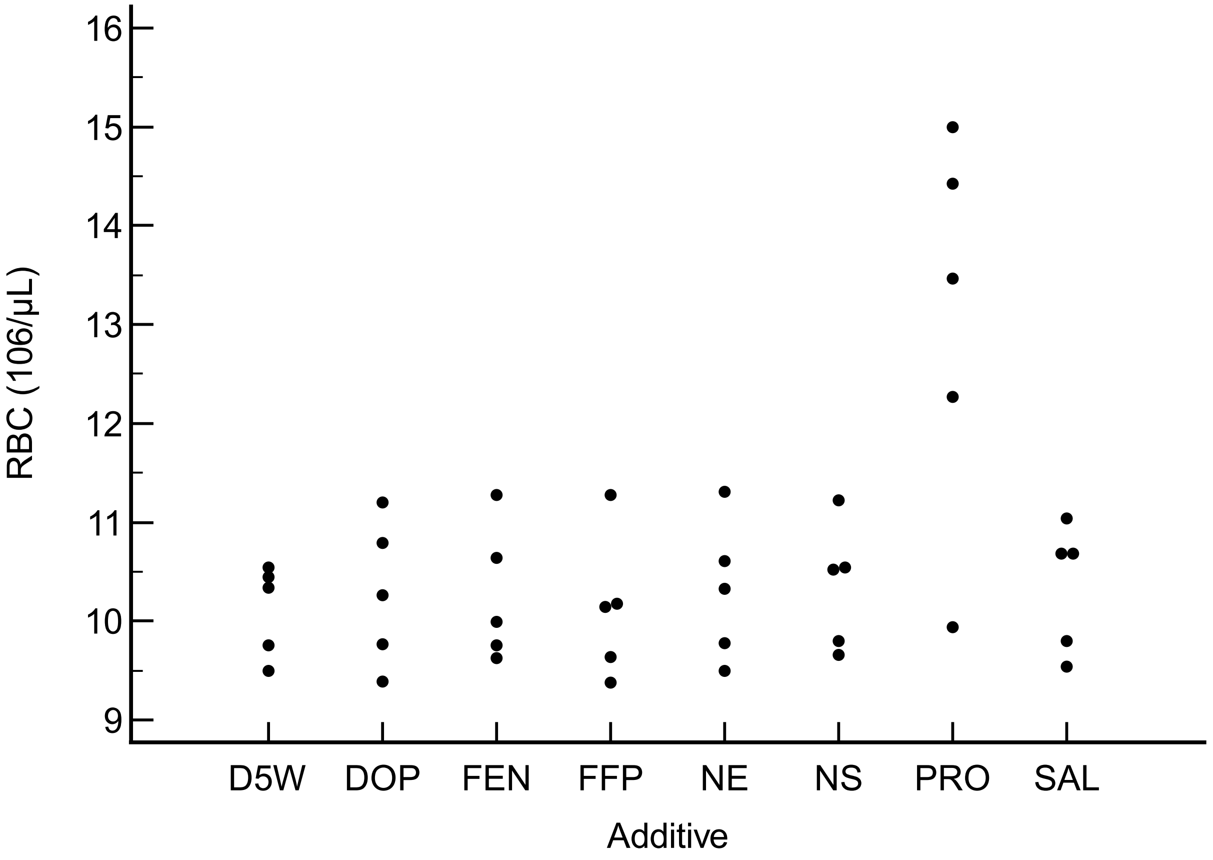

Condition: >28 day old blood, 3 min incubation

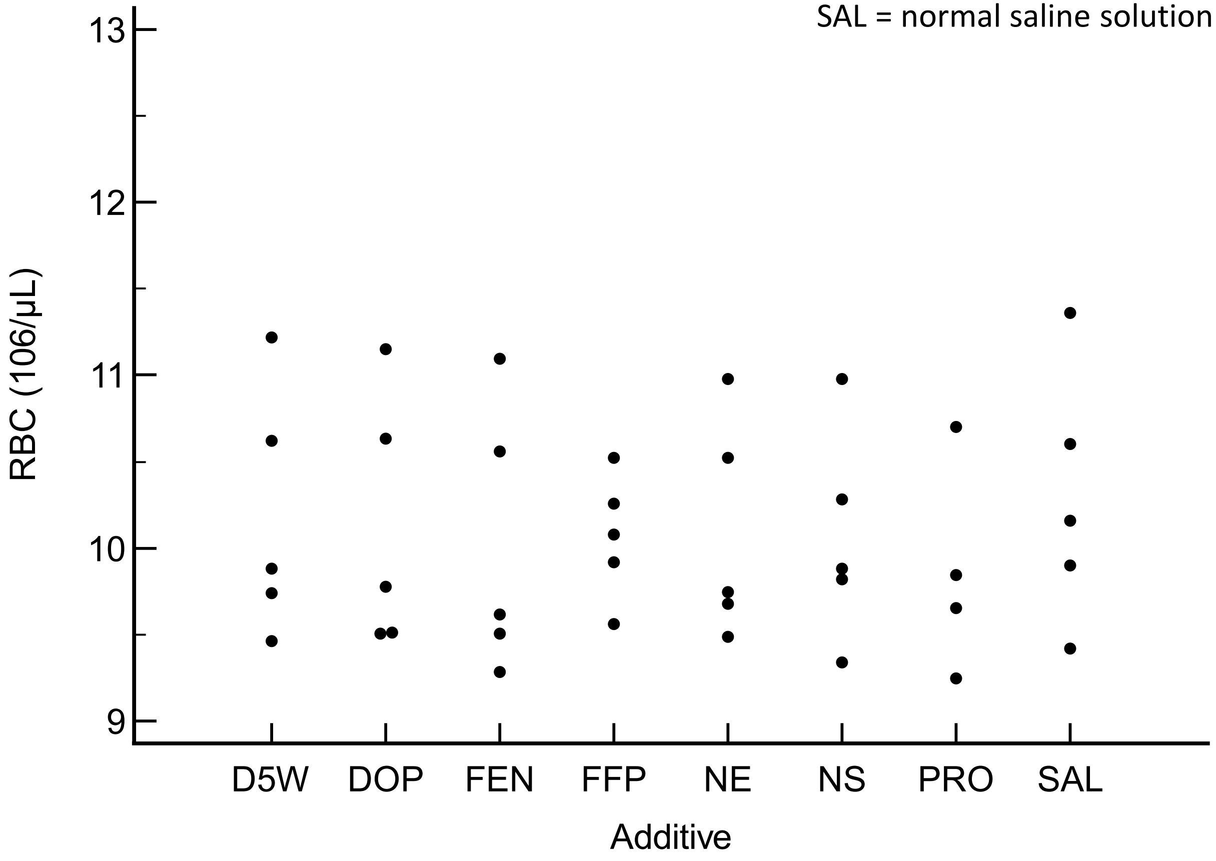

Condition: >28 day old blood, 30 min incubation

HGBchemical

D5W = 5% dextrose
DOP = Dopamine
FEN = Fentanyl
FFP = Fresh frozen plasma
NE = Norepinephrine
NS = Normosol-R
PRO = Propofol
SAL = normal saline solution

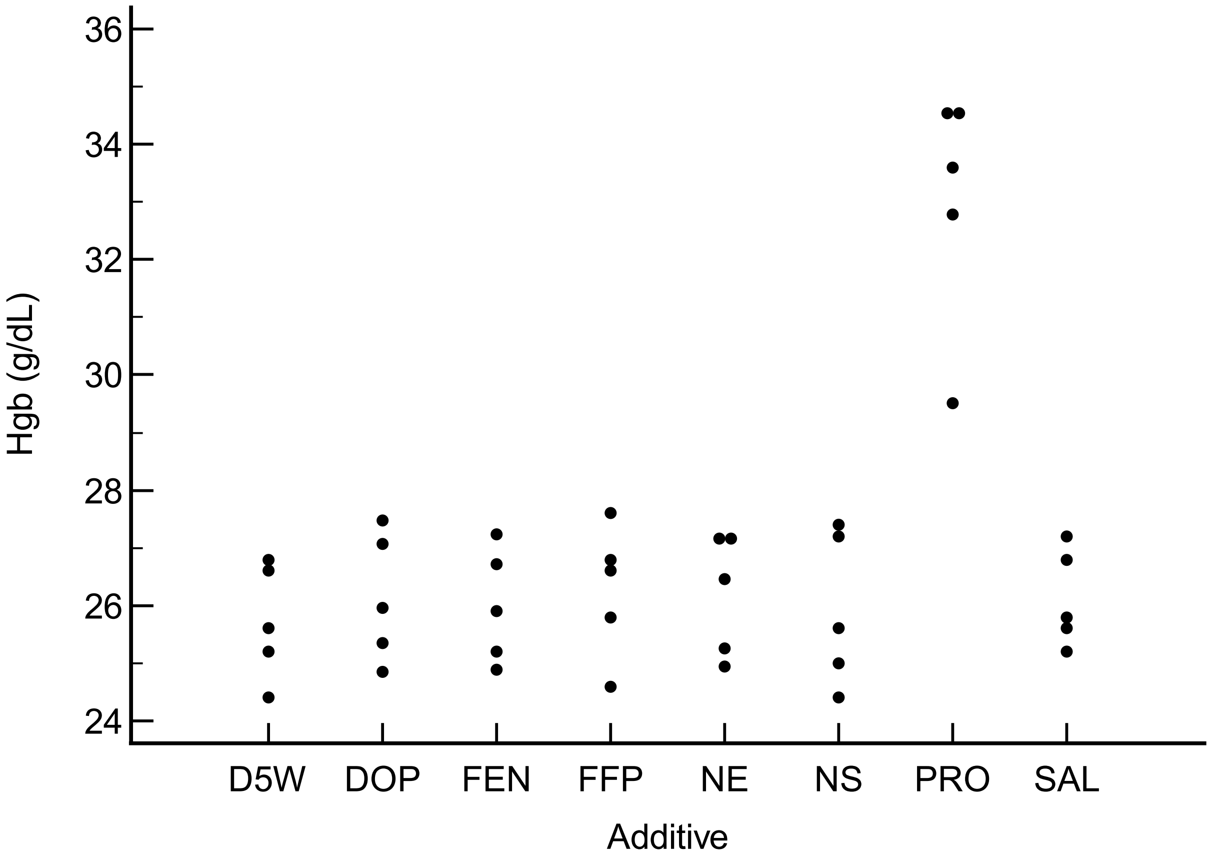

Condition: <7 day old blood, 3 min incubation

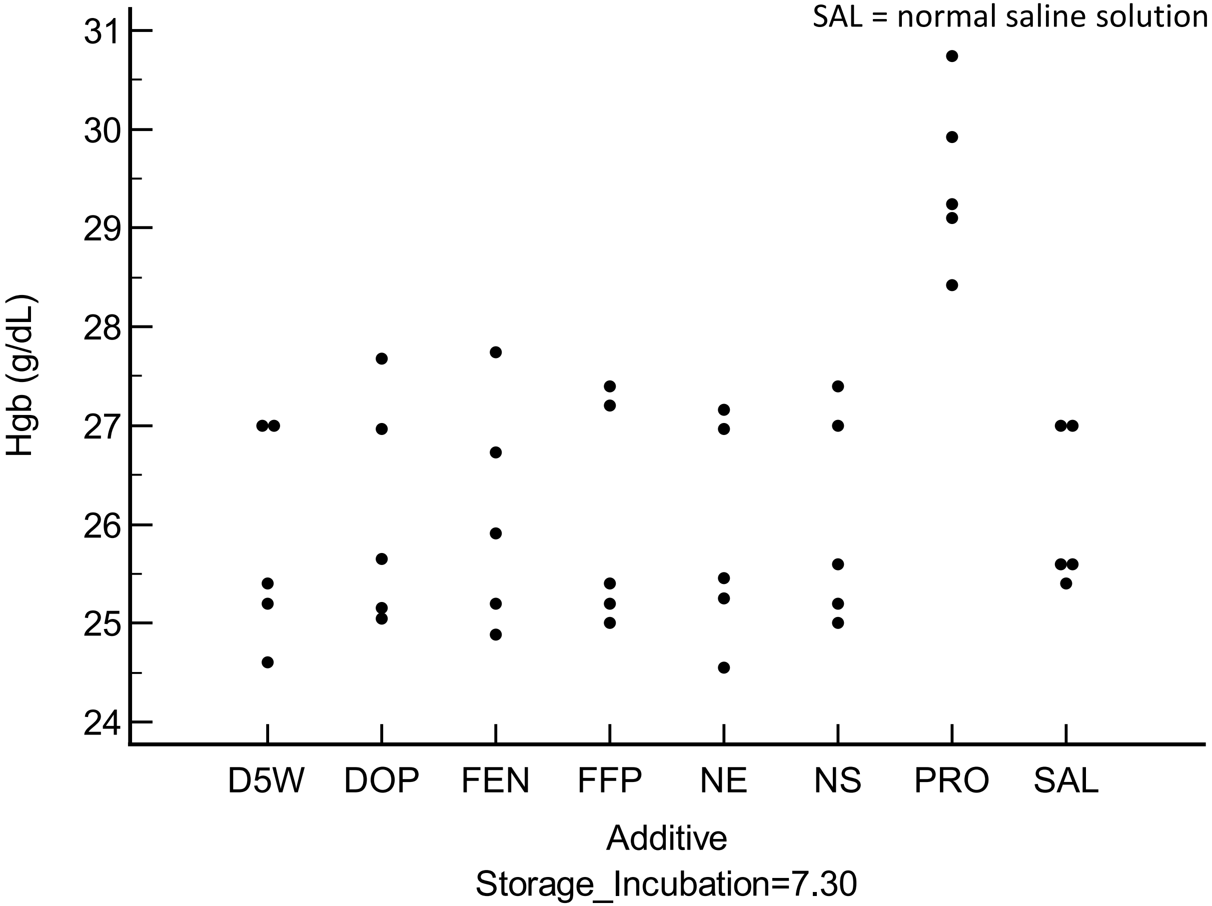

Condition: <7 day old blood, 30 min incubation

HGBchemical

D5W = 5% dextrose
DOP = Dopamine
FEN = Fentanyl
FFP = Fresh frozen plasma
NE = Norepinephrine
NS = Normosol-R
PRO = Propofol
SAL = normal saline solution

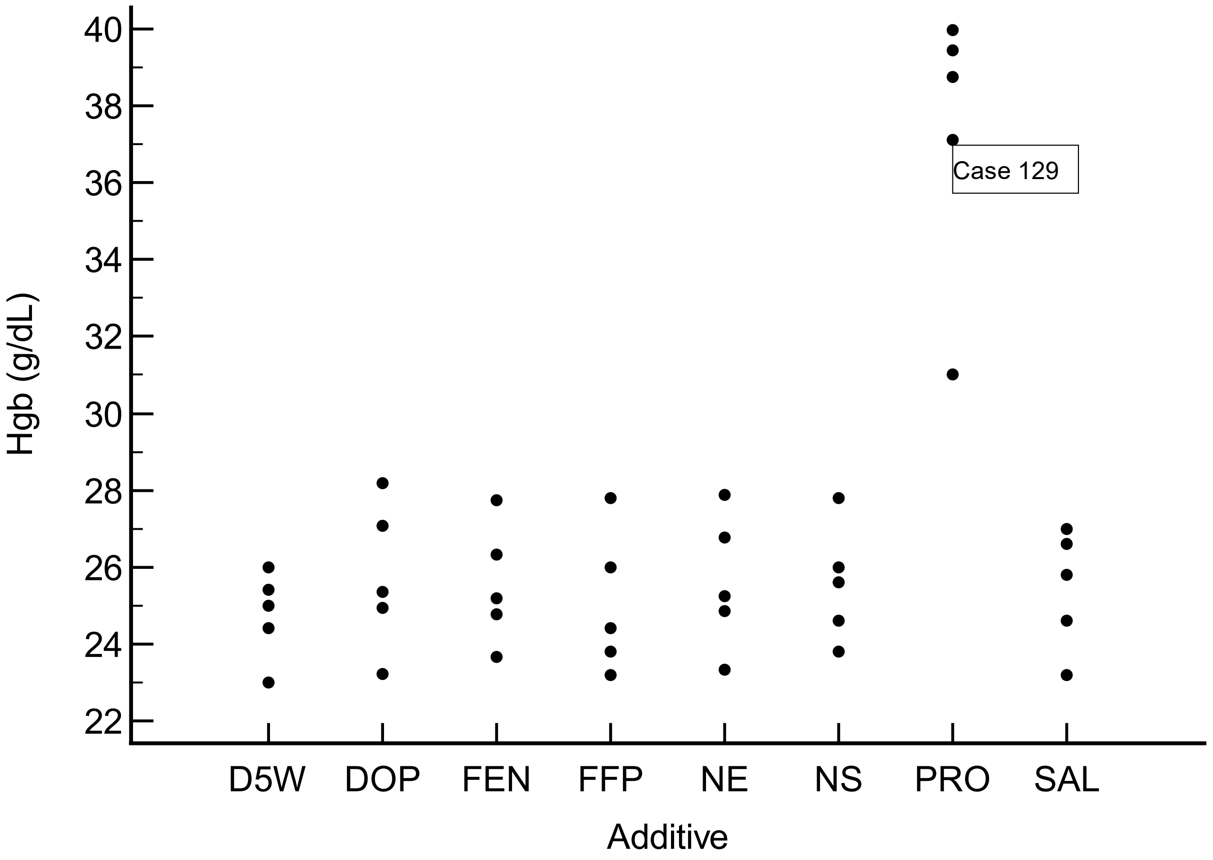

Condition: >28 day old blood, 3 min incubation

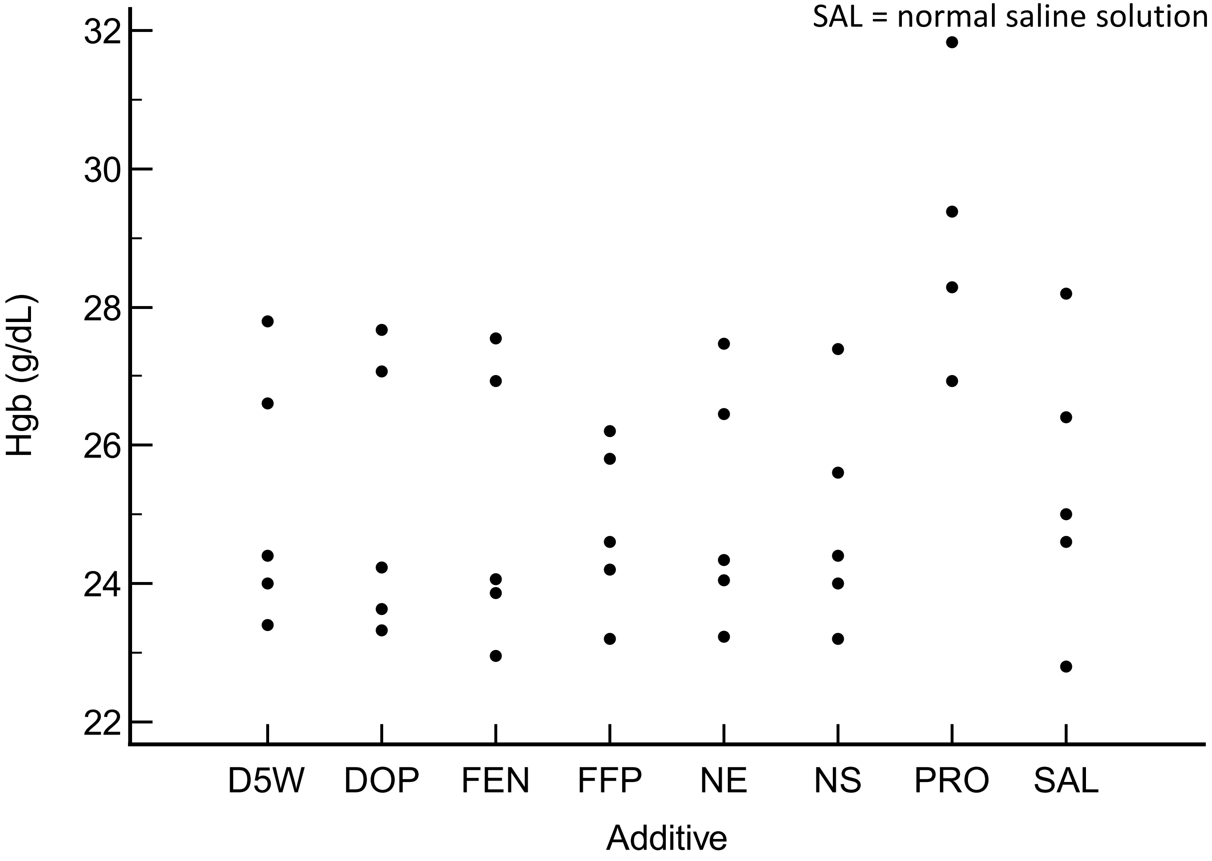

Condition: >28 day old blood, 30 min incubation

MCV

D5W = 5% dextrose
DOP = Dopamine
FEN = Fentanyl
FFP = Fresh frozen plasma
NE = Norepinephrine
NS = Normosol-R
PRO = Propofol
SAL = normal saline solution

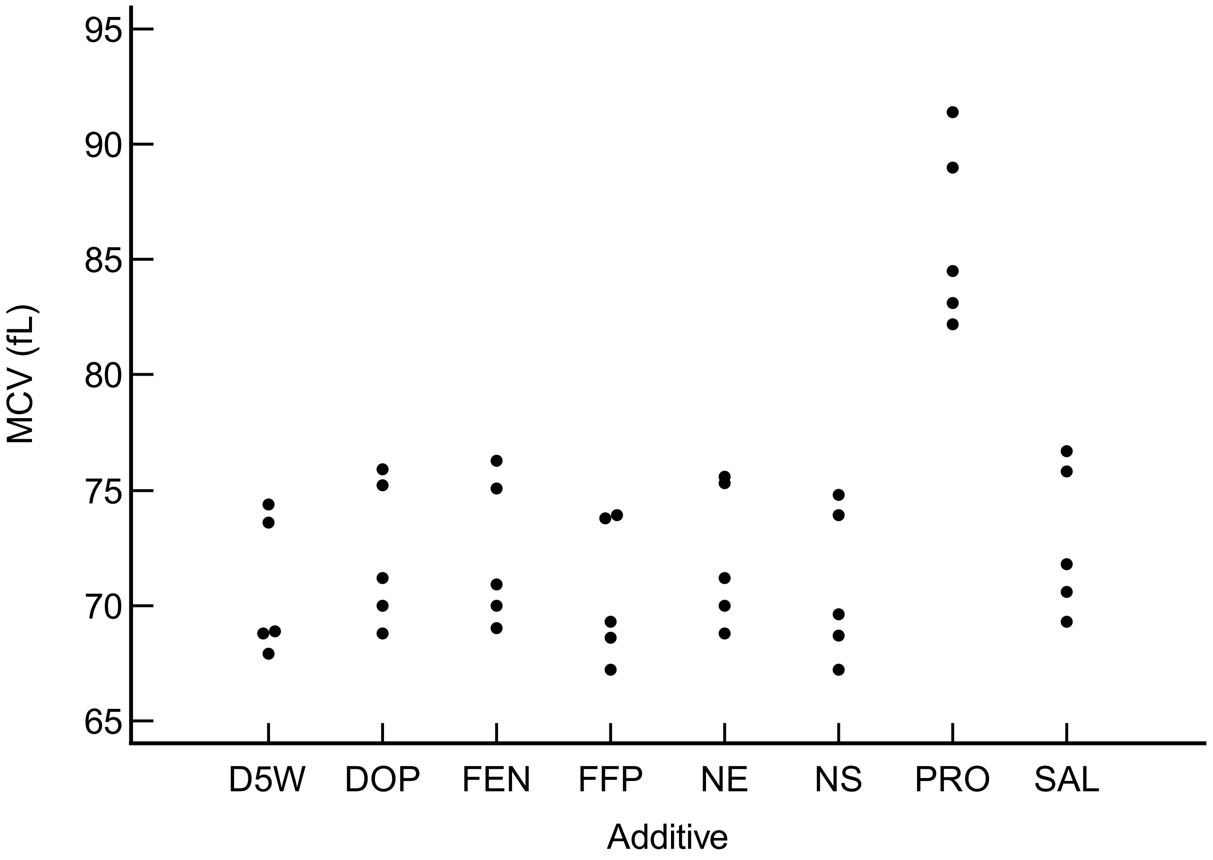

Condition: <7 day old blood, 3 min incubation

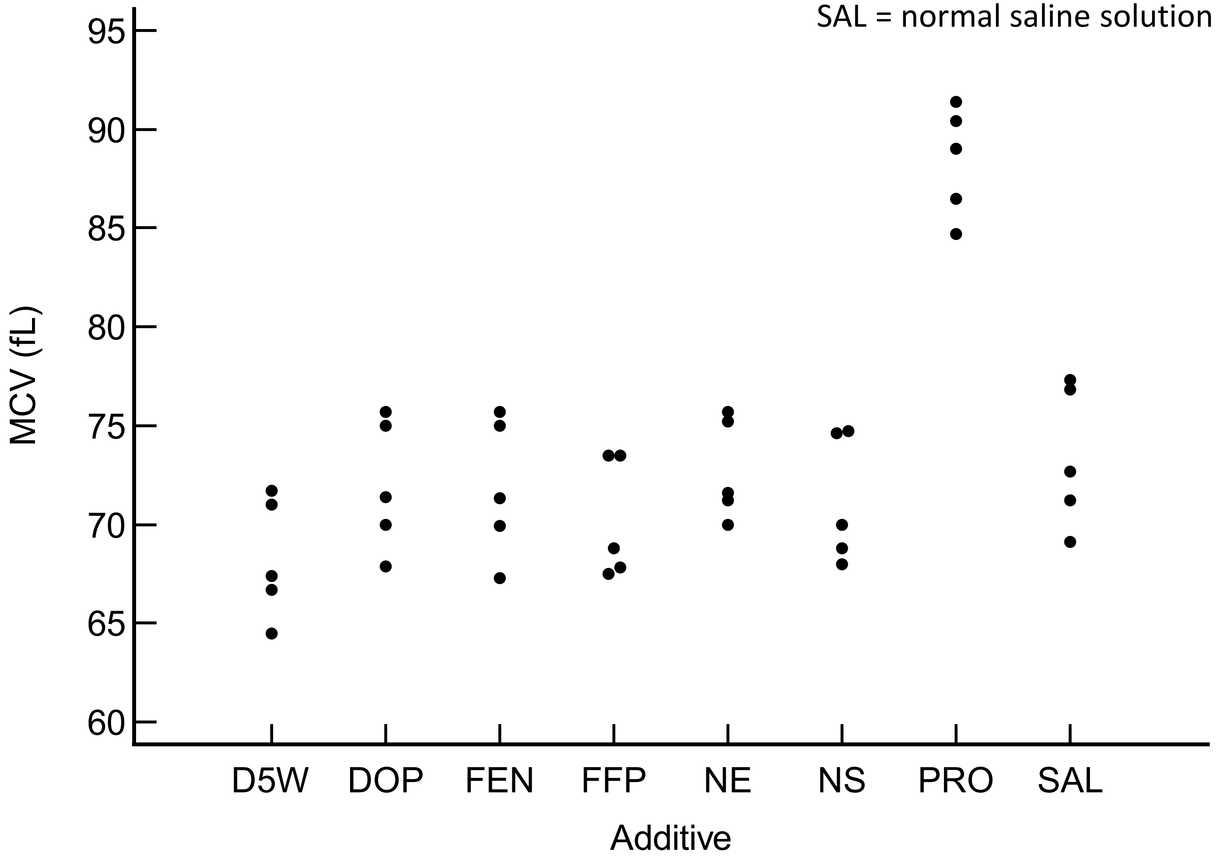

Condition: <7 day old blood, 30 min incubation

MCV

D5W = 5% dextrose
DOP = Dopamine
FEN = Fentanyl
FFP = Fresh frozen plasma
NE = Norepinephrine
NS = Normosol-R
PRO = Propofol
SAL = normal saline solution

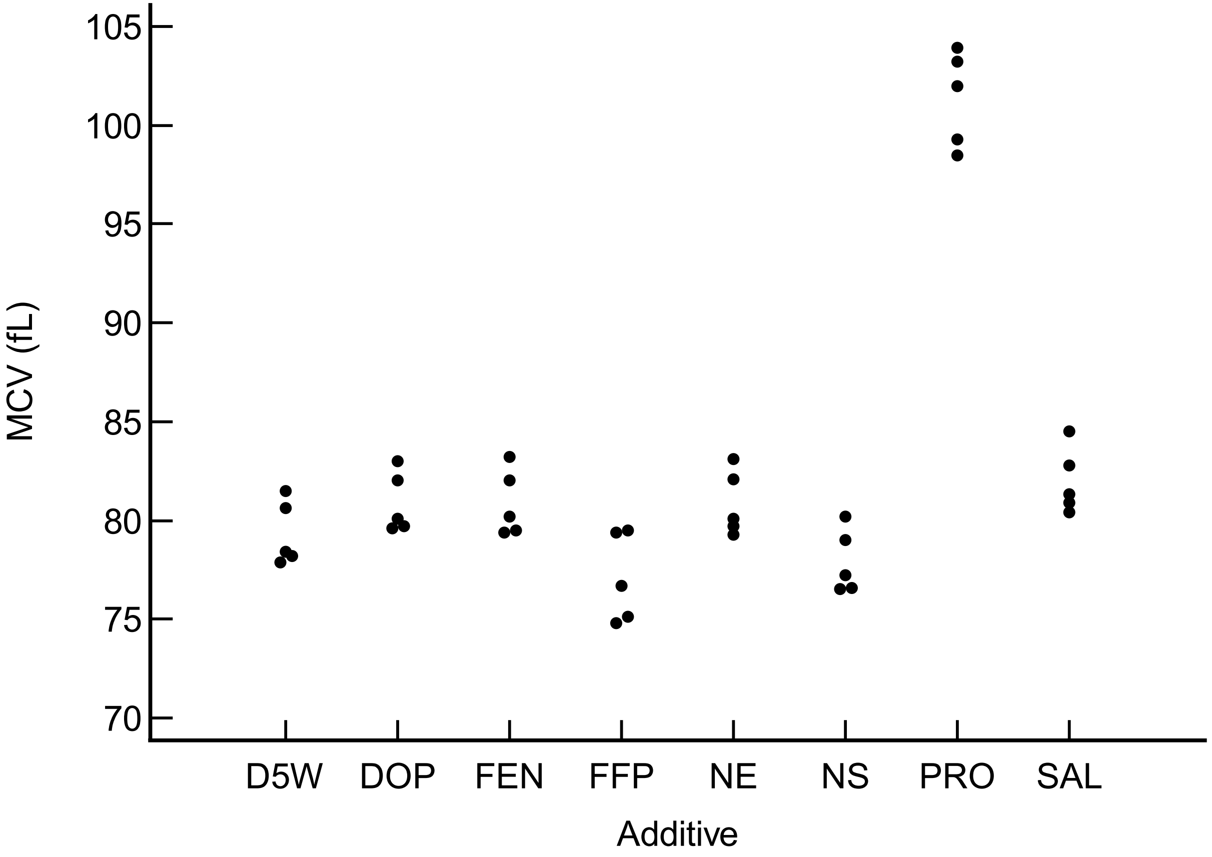

Condition: >28 day old blood, 3 min incubation

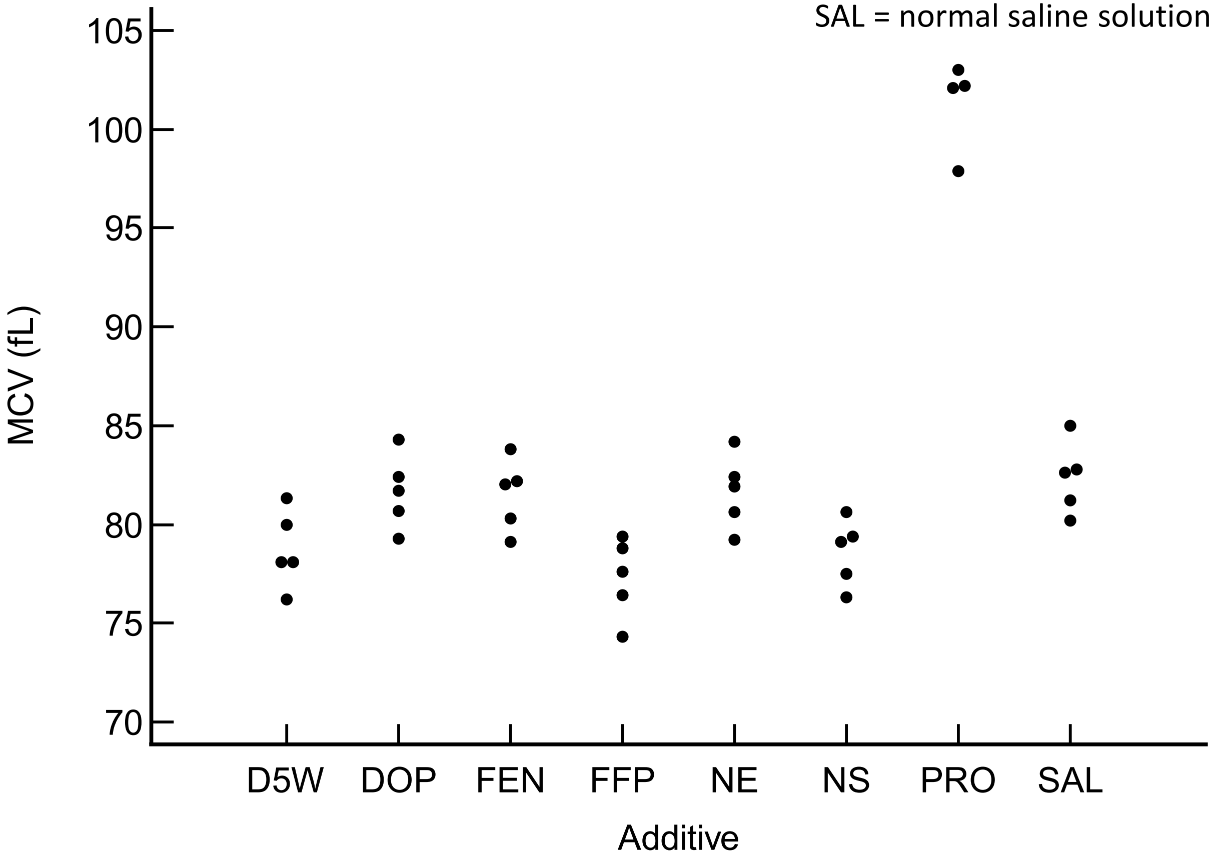

Condition: >28 day old blood, 30 min incubation

Cellular HGB

D5W = 5% dextrose
DOP = Dopamine
FEN = Fentanyl
FFP = Fresh frozen plasma
NE = Norepinephrine
NS = Normosol-R
PRO = Propofol
SAL = normal saline solution

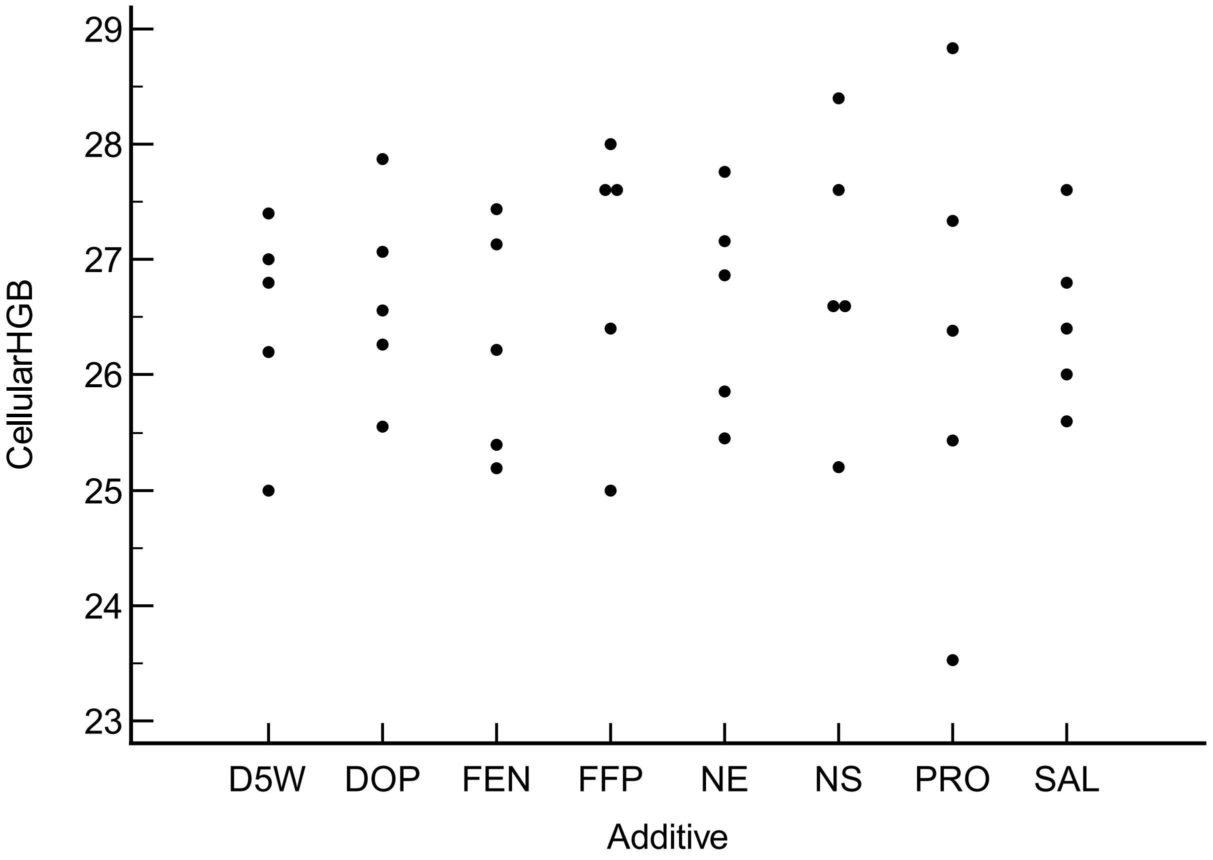

Condition: <7 day old blood, 3 min incubation

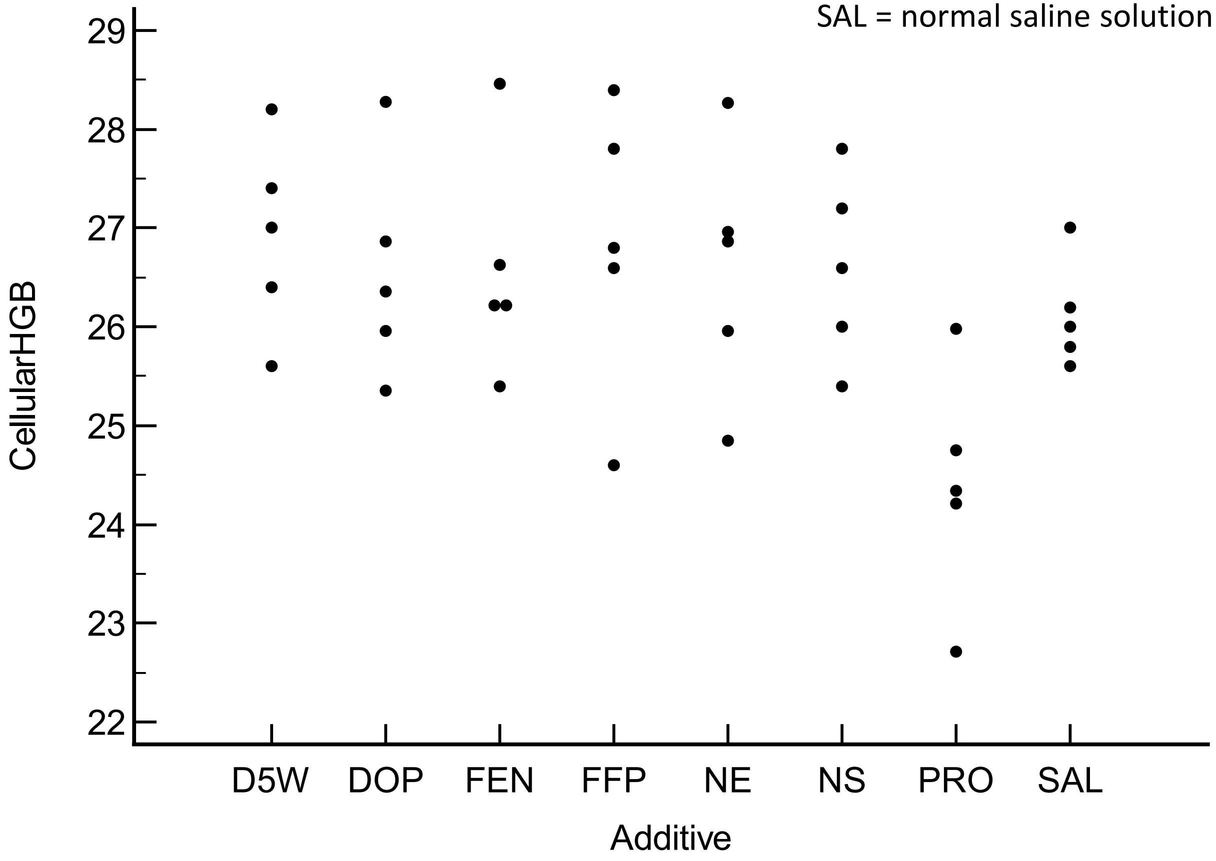

Condition: <7 day old blood, 30 min incubation

Cellular HGB

D5W = 5% dextrose
DOP = Dopamine
FEN = Fentanyl
FFP = Fresh frozen plasma
NE = Norepinephrine
NS = Normosol-R
PRO = Propofol
SAL = normal saline solution

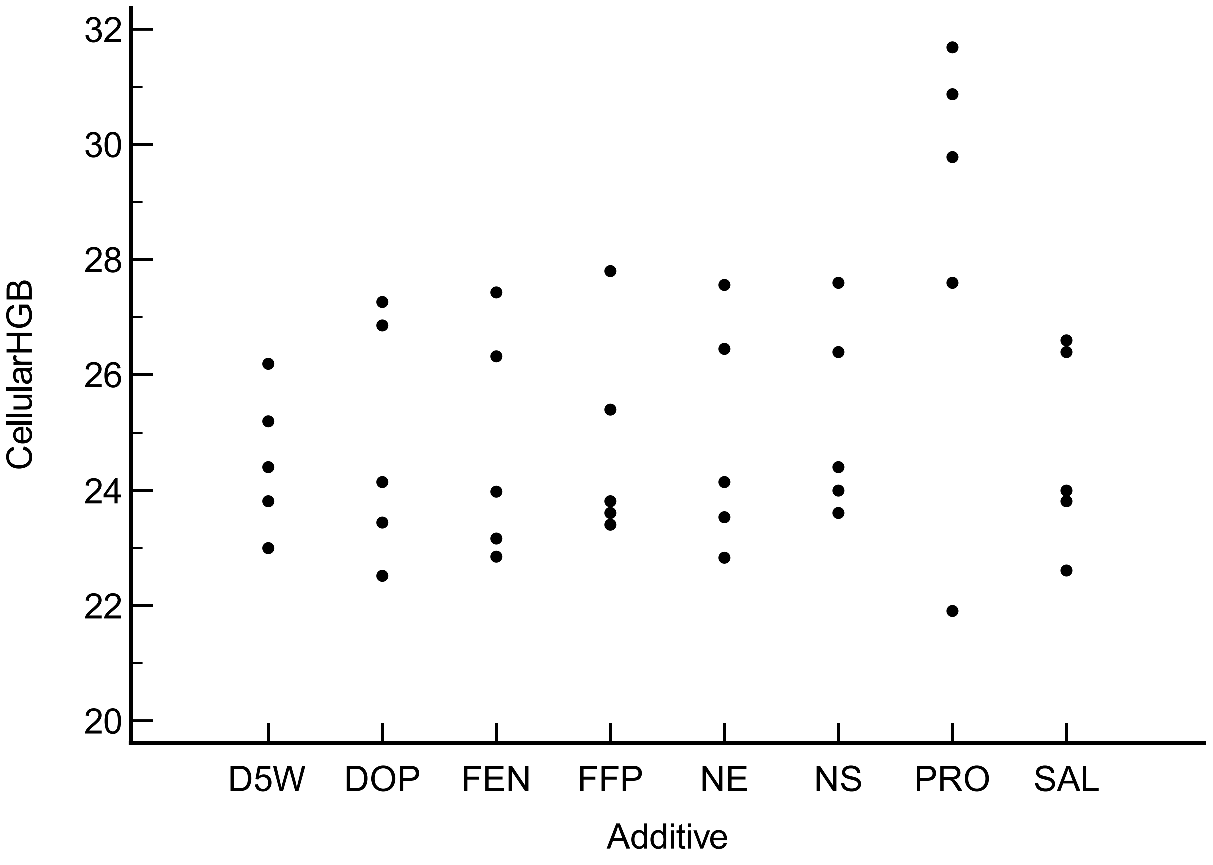

Condition: >28 day old blood, 3 min incubation

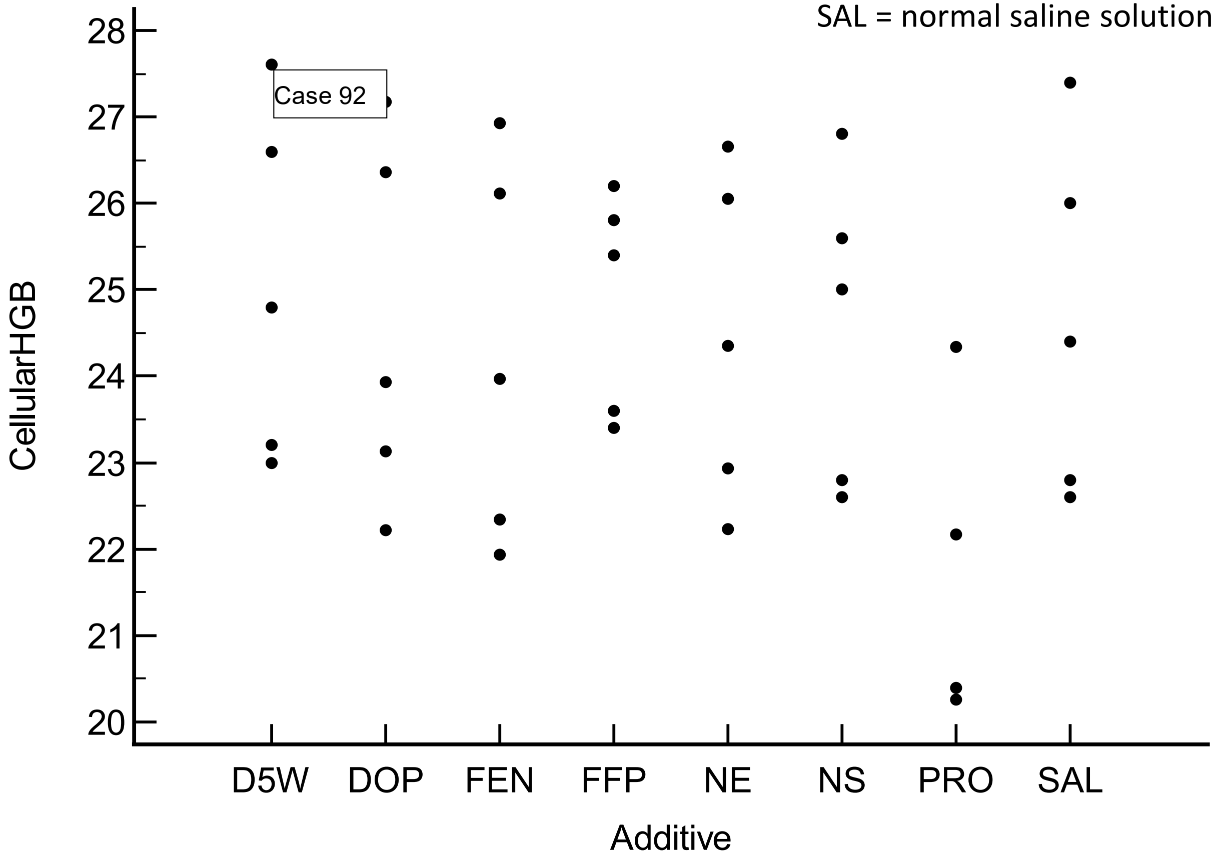

Condition: >28 day old blood, 30 min incubation

Platelet Count

D5W = 5% dextrose
DOP = Dopamine
FEN = Fentanyl
FFP = Fresh frozen plasma
NE = Norepinephrine
NS = Normosol-R
PRO = Propofol
SAL = normal saline solution

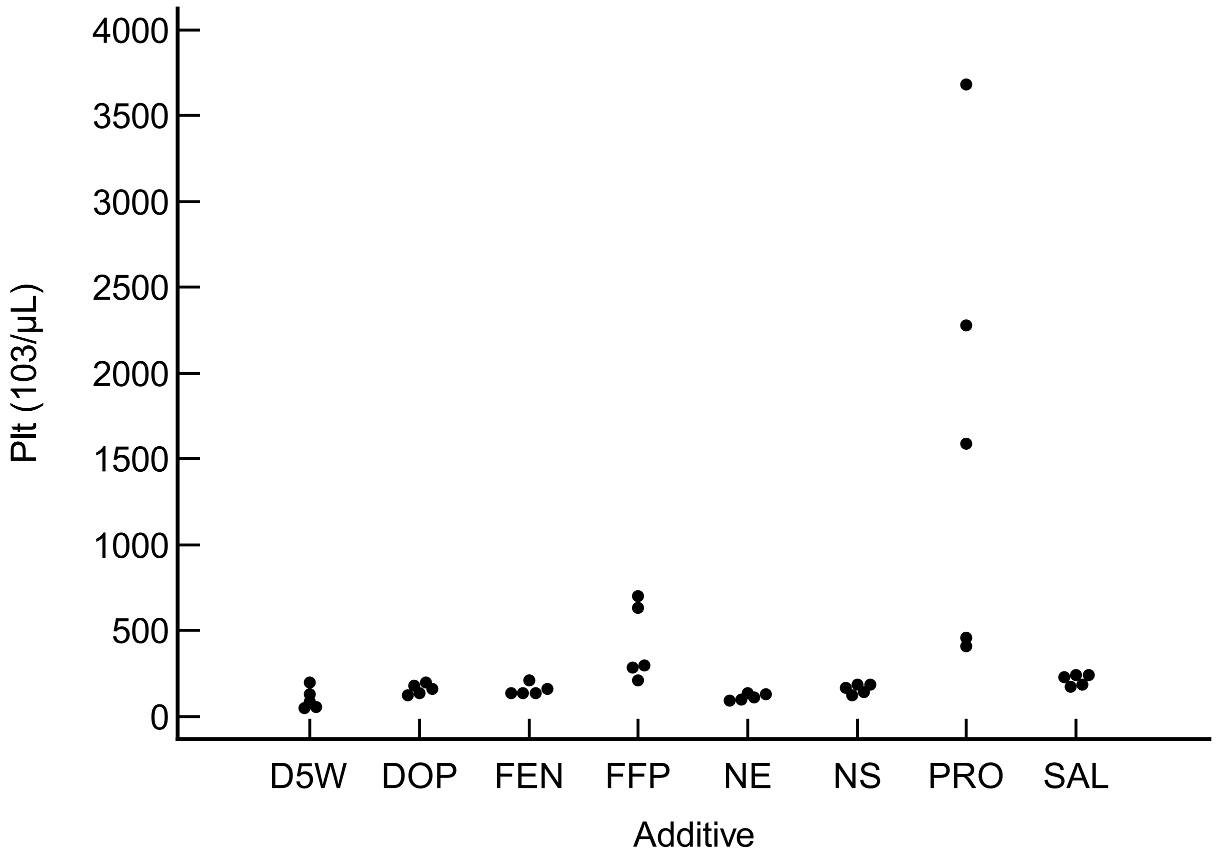

Condition: <7 day old blood, 3 min incubation

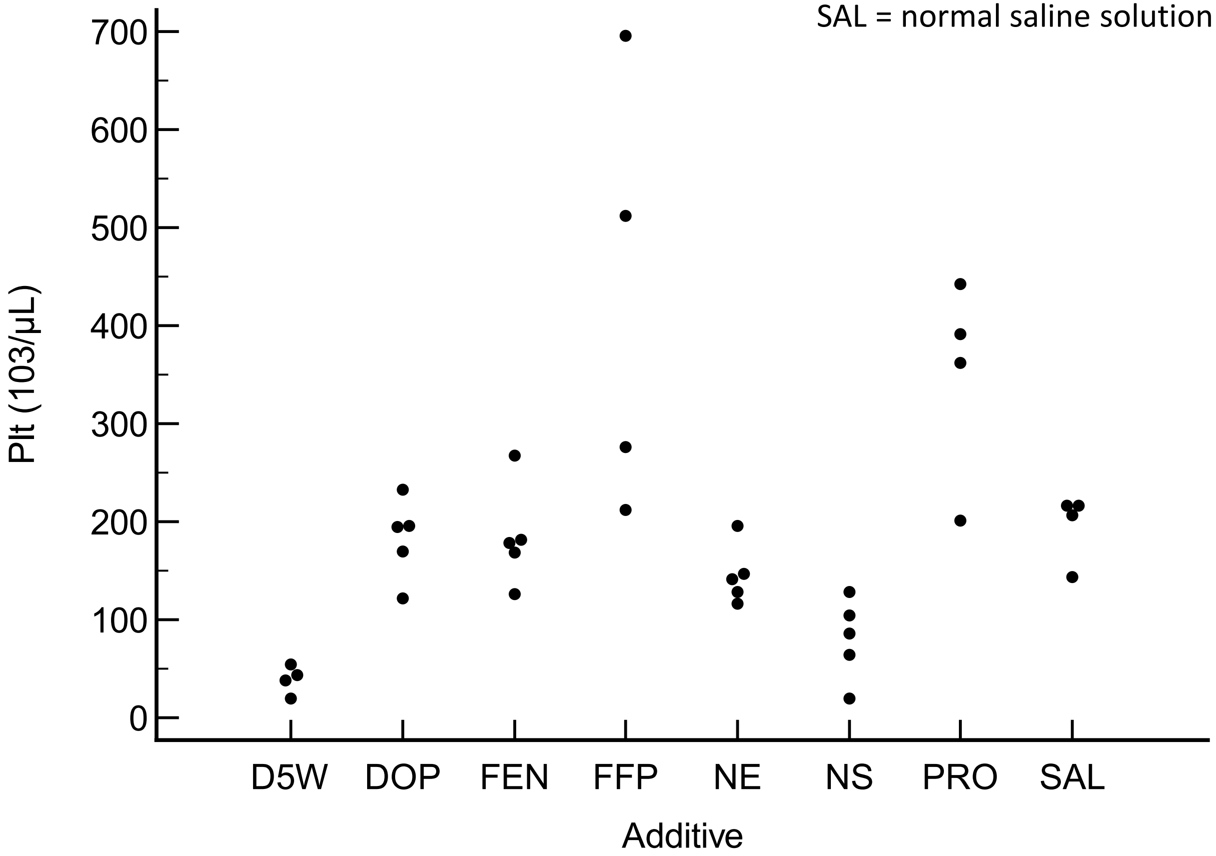

Condition: <7 day old blood, 30 min incubation

Platelet Count

D5W = 5% dextrose
DOP = Dopamine
FEN = Fentanyl
FFP = Fresh frozen plasma
NE = Norepinephrine
NS = Normosol-R
PRO = Propofol
SAL = normal saline solution

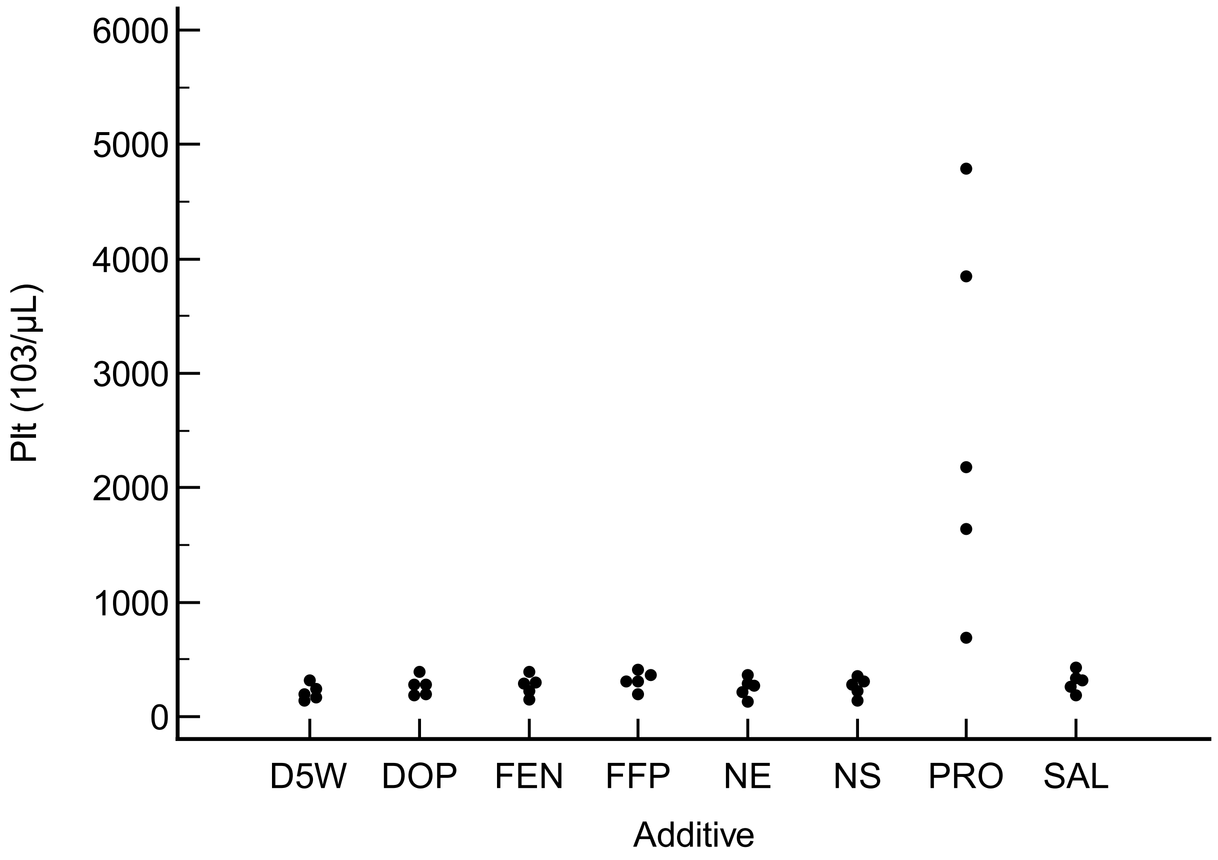

Condition: >28 day old blood, 3 min incubation

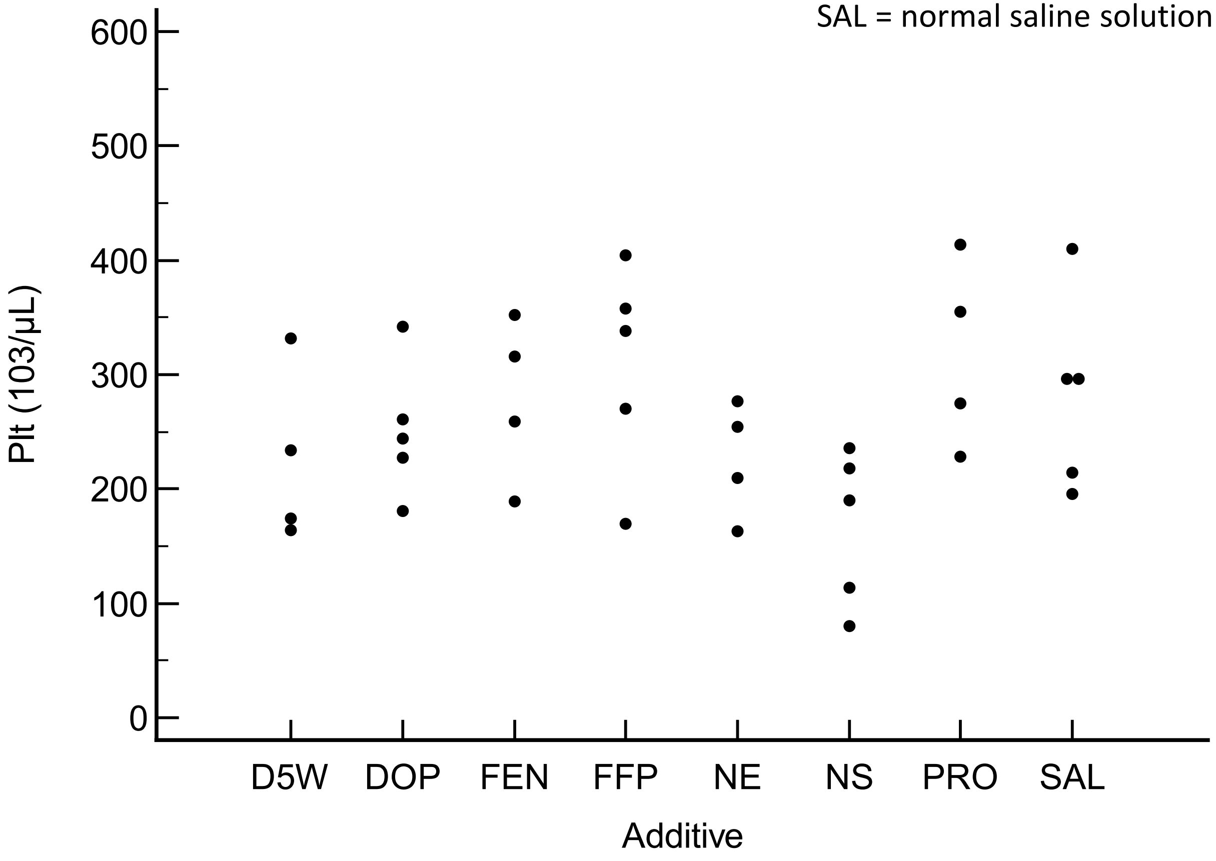

Condition: >28 day old blood, 30 min incubation

MPV

D5W = 5% dextrose
DOP = Dopamine
FEN = Fentanyl
FFP = Fresh frozen plasma
NE = Norepinephrine
NS = Normosol-R
PRO = Propofol
SAL = normal saline solution

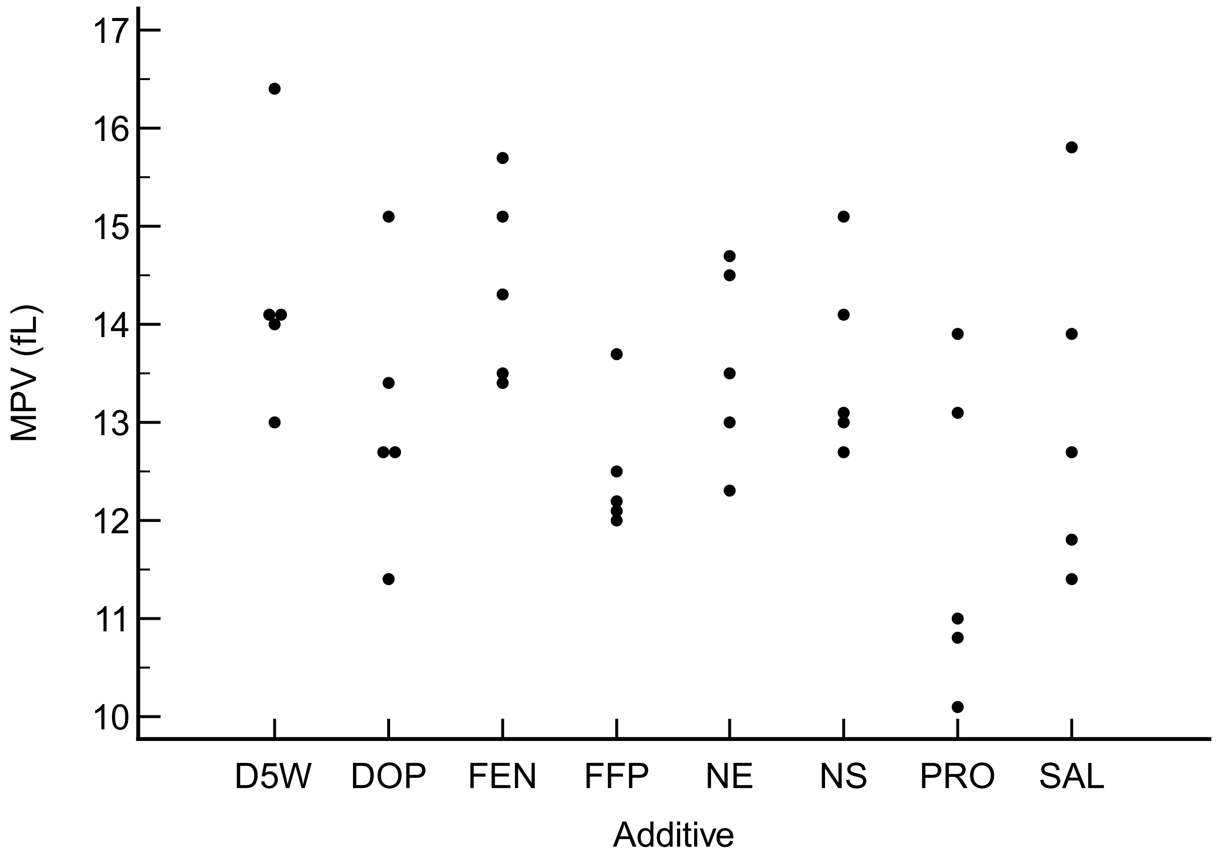

Condition: <7 day old blood, 3 min incubation

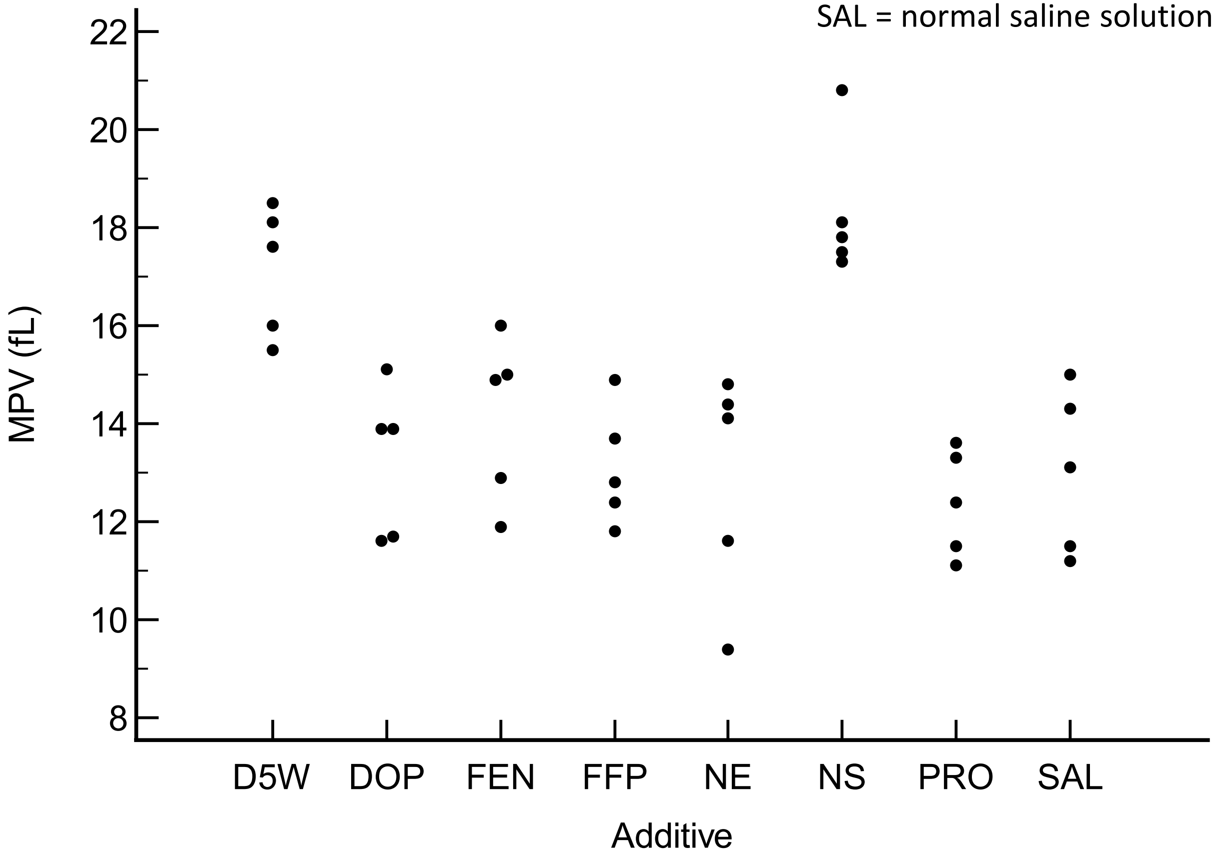

Condition: <7 day old blood, 30 min incubation

MPV

D5W = 5% dextrose
DOP = Dopamine
FEN = Fentanyl
FFP = Fresh frozen plasma
NE = Norepinephrine
NS = Normosol-R
PRO = Propofol
SAL = normal saline solution

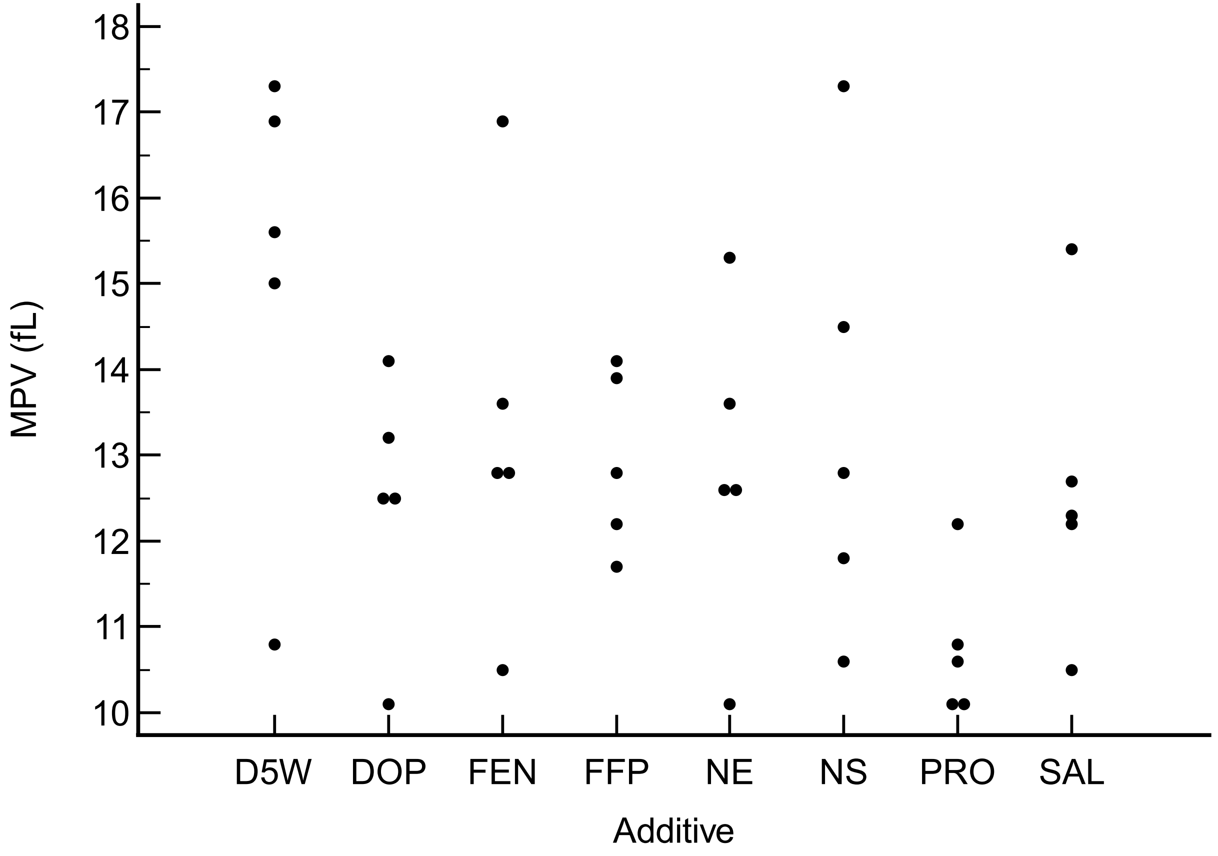

Condition: >28 day old blood, 3 min incubation

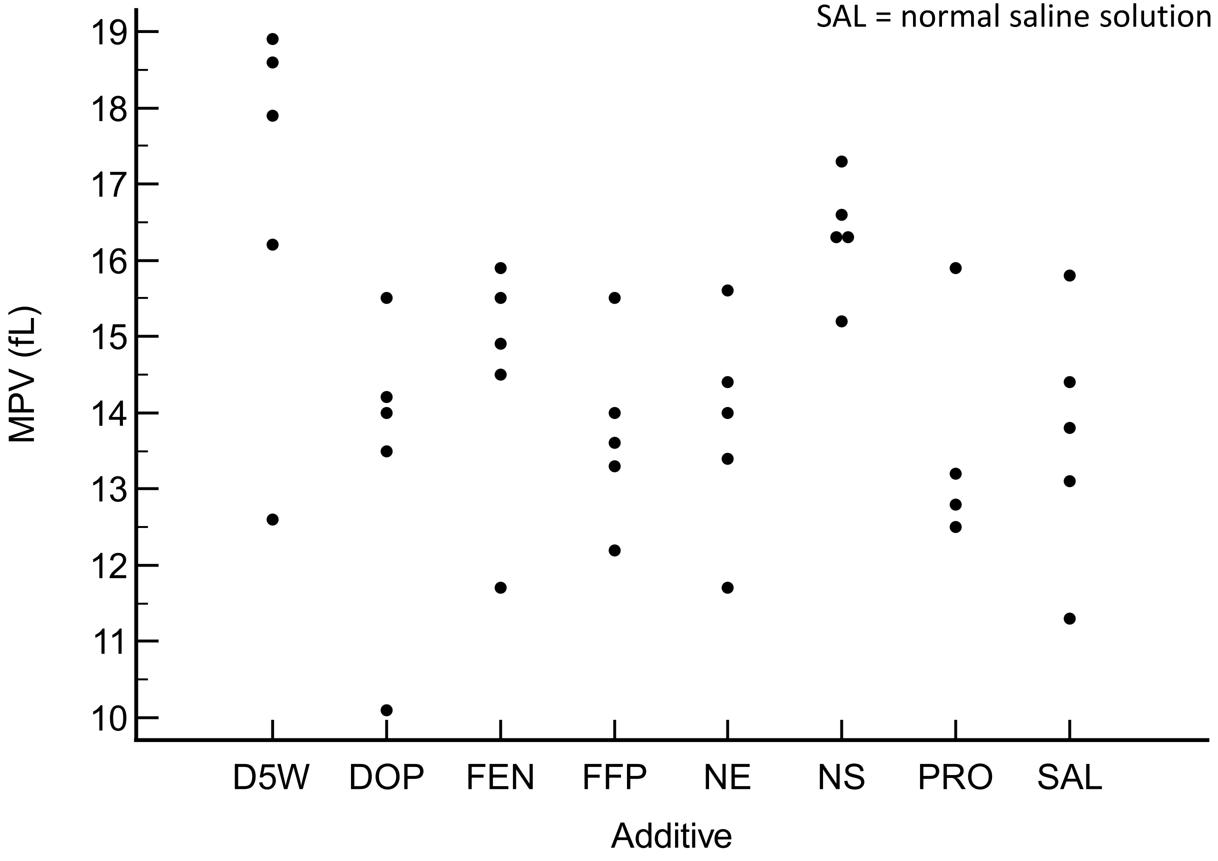

Condition: >28 day old blood, 30 min incubation

MPC

D5W = 5% dextrose
DOP = Dopamine
FEN = Fentanyl
FFP = Fresh frozen plasma
NE = Norepinephrine
NS = Normosol-R
PRO = Propofol
SAL = normal saline solution

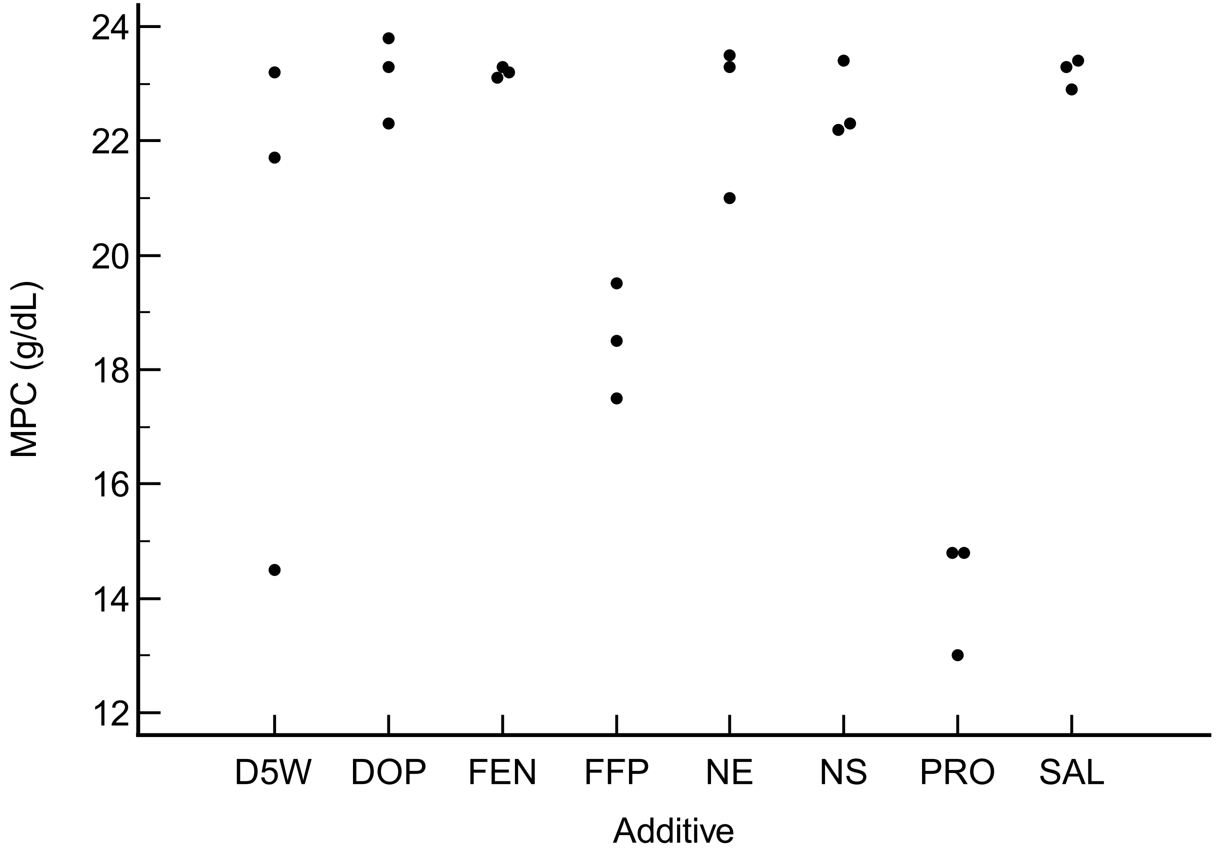

Condition: <7 day old blood, 3 min incubation

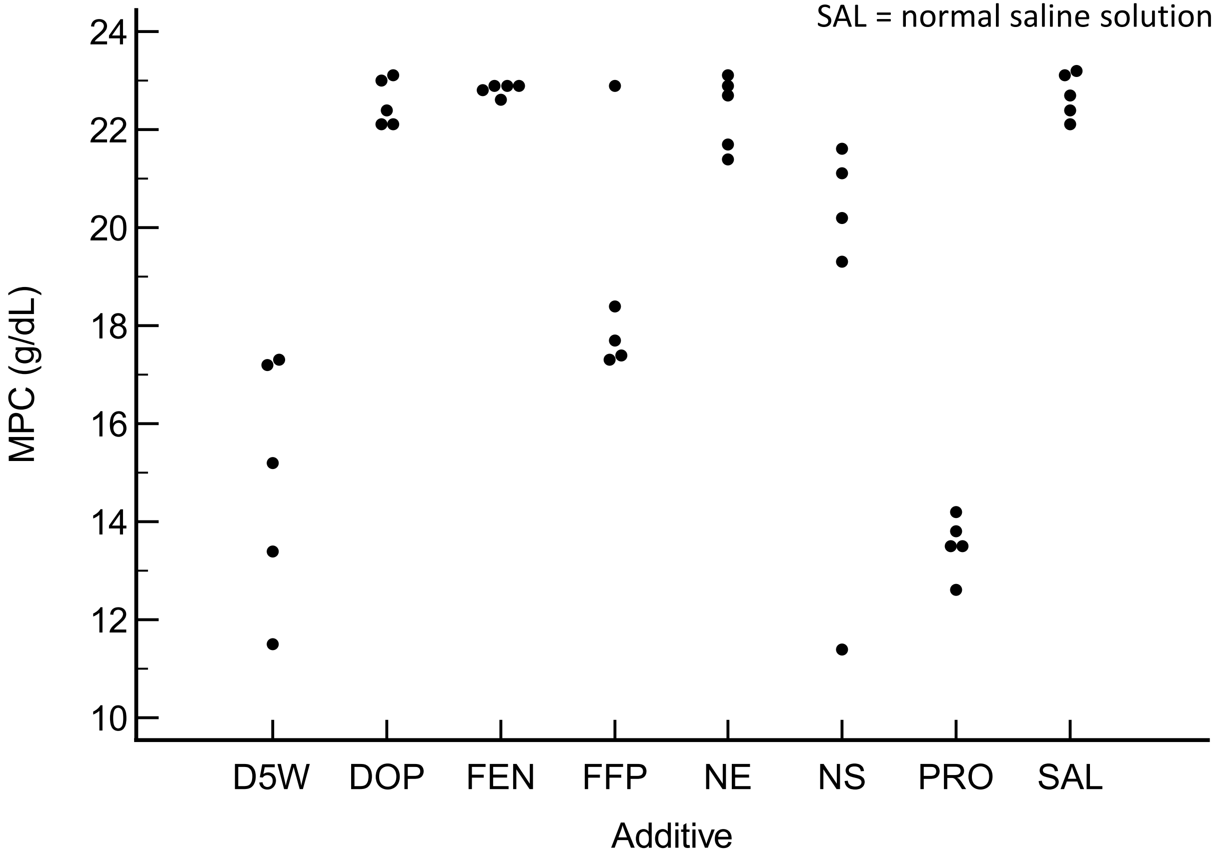

Condition: <7 day old blood, 30 min incubation

MPC

D5W = 5% dextrose
DOP = Dopamine
FEN = Fentanyl
FFP = Fresh frozen plasma
NE = Norepinephrine
NS = Normosol-R
PRO = Propofol
SAL = normal saline solution

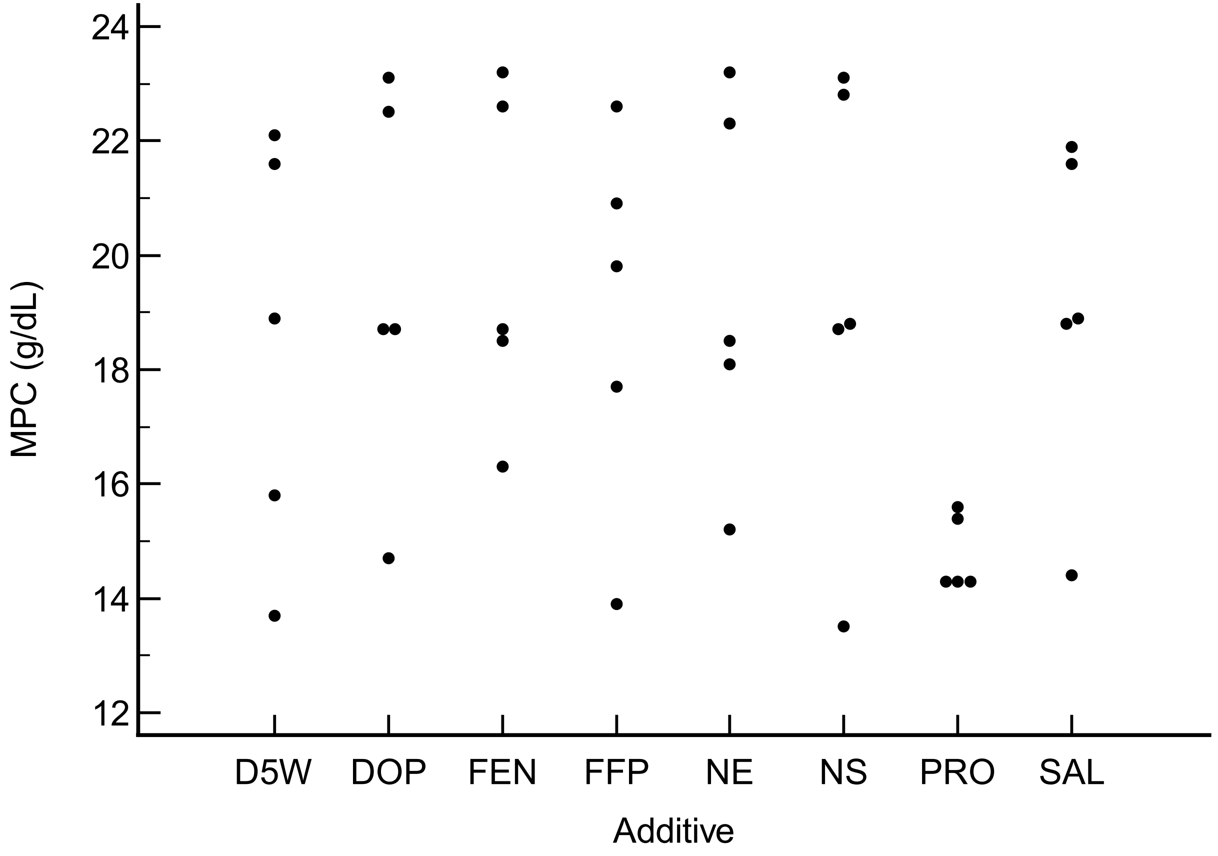

Condition: >28 day old blood, 3 min incubation

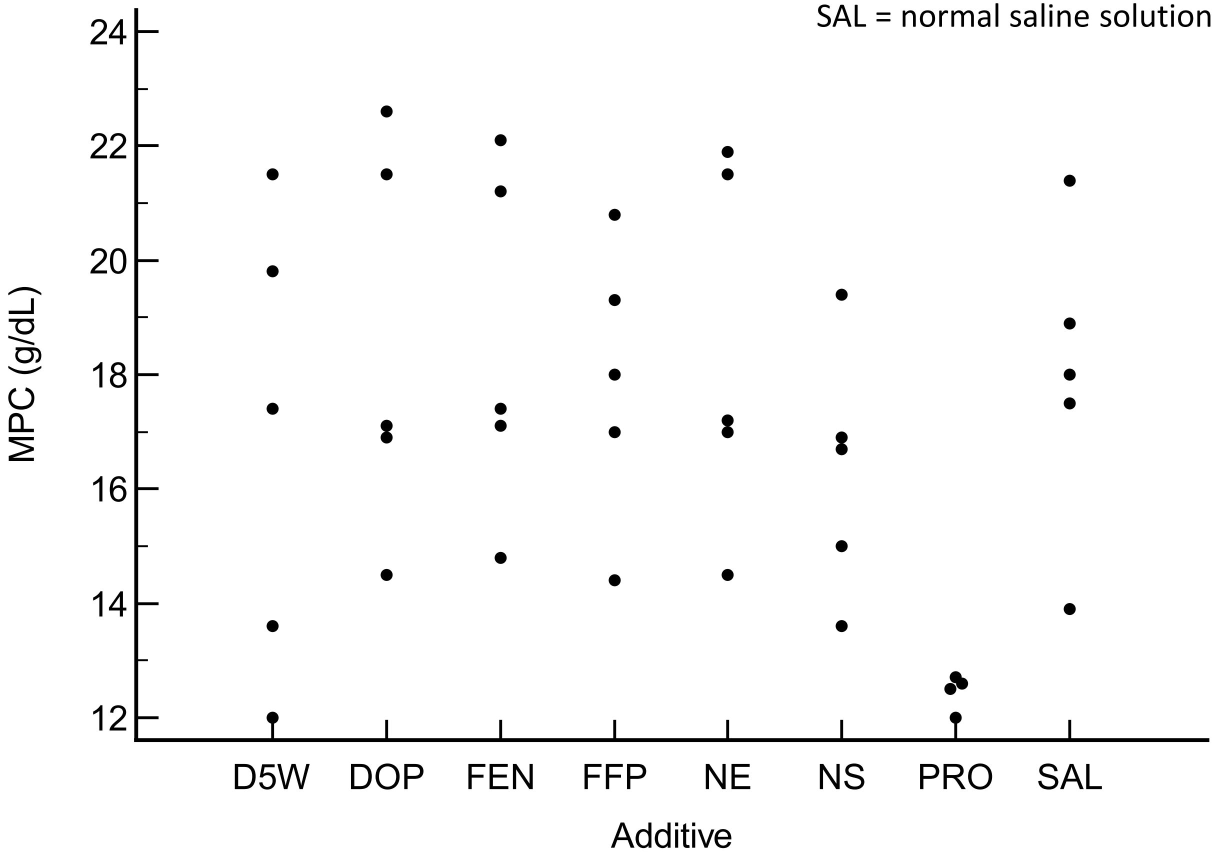

Condition: >28 day old blood, 30 min incubation
