# Supplementary material for: Effects of Coincubation With Crystalloids or Medications on Canine Packed Red Blood Cells: An In Vitro Evaluation
Source: J Vet Emerg Crit Care (San Antonio). 2026 May 14;36(3):308–16. doi: 10.1111/vec.70109 (PMC13350283; doi:10.1111/vec.70109)
Supplement: Supplementary file 2 — Supporting File 2: vec70109‐sup‐0002‐Table.docx. [file VEC-36-308-s001.docx]

Supplemental Table: CBC results for pRBC-free samples of FFP compared to propofol diluted in FFP (PRO/FFP) to the same concentration as used in the main experiments. Because the propofol sample was diluted as 360µL of propofol into 1000µL of FFP, these samples contained only 73.5% plasma. Therefore, the FFP values in the table are corrected for the comparison by multiplying each result by 0.735. Nonstandard abbreviations: cHGB, cellular hemoglobin; FFP, fresh frozen plasma; HGB, hemoglobin; PLT, platelet concentration.

|  | **Age of FFP (days)** | **RBC (x10^12^/L)** | **HGB (g/dL)** | **cHGB (g/dL)** | **PLT (x10^9^/L)** | **WBC (x10^9^/L)** |
| --- | --- | --- | --- | --- | --- | --- |
| **FFP x 0.735** | < 7 | 0.007 | 0.074 | 0 | 23.52 | 0.007 |
| **Propofol/FFP** | < 7 | 0.01 | 2.2 | 0 | 41 | 0.03 |
| **Absolute difference** | < 7 | 0.003 | 2.13 | 0 | 17.48 | 0.02 |
| **Percentage increase** | < 7 | 36% | 2893% | 0% | 74% | 308% |
| **FFP x 0.735** | > 28 | 0.015 | 0.074 | 0.07 | 39.690 | 0.029 |
| **Propofol/FFP** | > 28 | 0.15 | 12.9 | 0.3 | 487 | 0.13 |
| **Absolute difference** | > 28 | 0.14 | 12.83 | 0.23 | 447.31 | 0.10 |
| **Percentage increase** | > 28 | 920% | 17451% | 0% | 1127% | 342% |
